# DNA polymerase beta connects tumorigenicity with the circadian clock in liver cancer through the epigenetic demethylation of *Per1*

**DOI:** 10.1038/s41419-024-06462-7

**Published:** 2024-01-20

**Authors:** Siyu Chen, Wenxiang Zhang, Xiao Li, Zhengyu Cao, Chang Liu

**Affiliations:** 1 State Key Laboratory of Natural Medicines and School of Life Science and Technology, China Pharmaceutical University, Nanjing, 211198 Jiangsu China; 2 Department of Pathology, First Affiliated Hospital with Nanjing Medical University, Nanjing, 210029 Jiangsu China; 3 Jiangsu Provincial Key Laboratory for TCM Evaluation and Translational Development, School of Traditional Chinese Pharmacy, China Pharmaceutical University, Nanjing, 211198 Jiangsu China; 4 Chongqing Innovation Institute of China Pharmaceutical University, Chongqing, 401135 China

**Keywords:** DNA methylation, Liver cancer

## Abstract

The circadian-controlled DNA repair exhibits a strong diurnal rhythm. Disruption in circadian clock and DNA repair is closely linked with hepatocellular carcinoma (HCC) progression, but the mechanism remains unknown. Here, we show that polymerase beta (POLB), a critical enzyme in the DNA base excision repair pathway, is rhythmically expressed at the translational level in mouse livers. Hepatic *POLB* dysfunction dampens clock homeostasis, whereas retards HCC progression, by mediating the methylation of the 4th CpG island on the 5′UTR of clock gene *Per1*. Clinically, POLB is overexpressed in human HCC samples and positively associated with poor prognosis. Furthermore, the hepatic rhythmicity of POLB protein expression is orchestrated by Calreticulin (CALR). Our findings provide important insights into the molecular mechanism underlying the synergy between clock and food signals on the POLB-driven BER system and reveal new clock-dependent carcinogenetic effects of POLB. Therefore, chronobiological modulation of POLB may help to promote precise interventions for HCC.

## Introduction

The genome is sensitive to a variety of endogenous and exogenous DNA-damaging agents that modify DNA bases and deoxyribose phosphate (dRP) groups, as well as directly break DNA backbone [1]. To repair these damages, organisms have evolved multiple DNA repair mechanisms, which are essential to preserve the integrity of genomic DNA [1]. Malfunctions in DNA repair systems lead to a series of serious consequences, including heightened sensitivity to environmental changes, increased susceptibility to tumorigenesis, and accelerated aging [2].

As a key factor, POLB is essentially required for all forms of base excision repair (BER). It is a 39 kDa protein with both DNA polymerase and dRP lyase activities, which are responsible for the incorporation of new deoxyribonucleotides and the removal of the dRP group, respectively [3, 4]. In addition, POLB interacts with many partner proteins, such as AP endonuclease 1 (APEX1), flap endonuclease 1 (FEN1), and proliferating cell nuclear antigen (PCNA) [5]. These interactions are important for the coordination of the BER process and ensure its efficiency and accuracy. Given the importance of POLB in the BER machinery, abnormalities in its expression and activity are closely correlated with tumorigenesis. For example, POLB was found to be overexpressed in approximately one-third of a diverse range of matched tumor samples [4]. Aberrant high levels of POLB in uterus and ovary cancer samples could potentially destabilize other cellular processes and eventually lead to tumorigenesis or progression [6]. On the other hand, *POLB* mutations with lower enzymatic activities have also been detected in most types of human cancers, including colorectal tumor, gastric carcinoma and prostate cancers [5]. Such *POLB* variants may result in BER deficiencies and increase the risk of cancer development. In particular, three germline polymorphic variants of POLB with single amino acid substitution have been identified in the human population. These variants include R137Q, P242R and Q8R variants [7]. However, their roles in cancer pathogenesis are still unknown. For example, the P242R mutation results in an elevation of chromosomal aberrations and cellular transformation, ultimately leading to an enhanced predisposition to cancer [8]. Individuals carrying this allele are present in Eastern European populations, and heterozygous patients have demonstrated decreased survival rates during their therapeutic periods for lung cancer or lymphoma treatment [9, 10]. More importantly, R137Q variant is of particular interest because the amino acid substitution of Arg by Gln leads to a net positive charge loss, potentially causing significant changes in the biochemical and physiological properties of the enzyme. The POLB R137Q variant has been shown to exhibit decreased DNA synthesis activity and impaired interaction with PCNA, further leading to cellular hypersensitivity to DNA-damaging agents, such as methyl methanesulfonate and N-methyl-N-nitrosourea, in Hela cells [7]. In addition, R137Q knock-in mice have been found to exhibit retarded embryo development and a high mortality rate [11]. Although R137 is a key amino acid site for proper POLB function in maintaining genomic DNA stability and physiological homeostasis, the causal relationship between the POLB R137Q mutant and cancer development remains unknown.

Of note, the risk of DNA damage is not constant throughout the day. Factors such as UV light, which potentially triggering DNA damage, vary significantly depending on the light/dark cycles induced by the Earth’s autorotation, thus leading to the fluctuation in the risk of DNA damage [12]. In fact, nearly all behavioral and physiological activities in organisms exhibit oscillations in response to diurnal changes of environmental light and food availability. This adaptation is controlled by the circadian clock system, which is based on interconnecting transcriptional-translational loops of clock genes/proteins that are ubiquitously expressed in cells [13]. In the core loop of the circadian clock system, the transcriptional activators of circadian locomotor output cycles kaput (CLOCK) and brain and muscle ARNT-like 1 (BMAL1) form heterodimers and activate the expression of repressors encoded by three Period (PER1-3) and two Cryptochrome (CRY1/2) genes. As the day progresses, protein complexes consisting of PER and CRY translocate into the nucleus and inhibit CLOCK/BMAL1-mediated transcription. Because of their instability, the protein and mRNA levels of these repressors rapidly decrease below the threshold required for autorepression, allowing for a new cycle to begin [14–16]. A growing body of epidemiologic data suggests that circadian clock disruption predisposes humans to cancer. For instance, certain lifestyle choices and occupations that disrupt the day-night rhythm have been linked to a higher incidence of colorectal carcinoma, breast cancer, lung cancer, and prostate cancer in humans [17–20]. Consistent with this, circadian dysfunction induces spontaneous hepatocarcinogenesis [21]. Conversely, cancer development also disrupts the circadian clock. At the molecular level, the circadian clock regulates various genes involved in important steps during tumorigenesis, such as cell cycle re-entry, proliferation, invasion, and metastasis [22]. Collectively, the circadian clock and cancer pathogenesis are tightly integrated and are reciprocally regulated.

Considering that perturbations in both the circadian rhythm and DNA repair are implicated in the development of hepatocellular carcinoma (HCC) [23, 24], while POLB serves as a crucial mediator of DNA synthesis and cellular susceptibility to DNA-damaging agents [25], we aimed to examine the involvement of POLB and the biological clock in the coordinated regulation of HCC pathophysiology. We found that POLB exhibited diurnal rhythmicity and was post-translationally regulated by Calreticulin (CALR) in a circadian manner. Hepatic POLB served as a critical component for maintaining circadian homeostasis and was clinically overexpressed in the HCC patients, driving cancer progression through demethylation of the 4th CpG island on the 5′UTR of clock gene *Per1*. Our findings furnish key insights into the molecular mechanism that drives the synergistic effects of clock and food signals on the POLB-driven BER system, and uncover the novel clock-linked carcinogenic effects of POLB in the liver. Therefore, chronobiological modulation of POLB may present a promising avenue for the precise interventions targeting HCC.

## Results

### Hepatic POLB responses to peripheral clocks and food signals

**Fig. 1:**
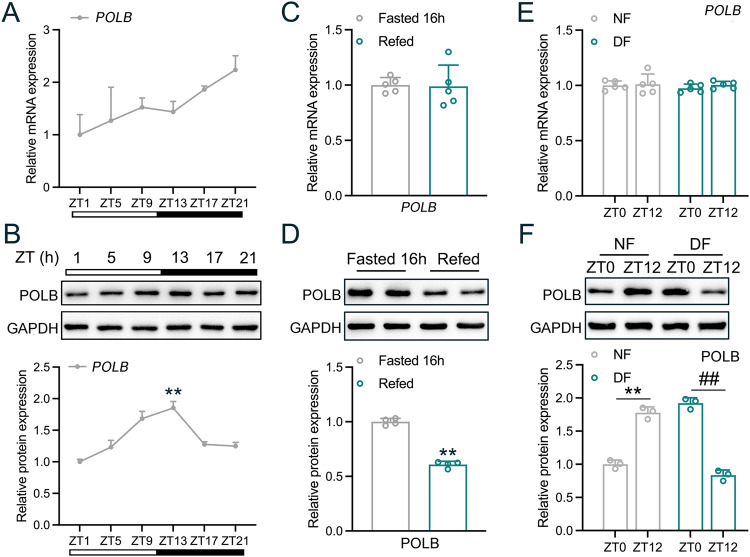
Hepatic POLB responses to peripheral clocks and food signals. RT-qPCR (**A**) and Western blot (**B**) analyses of POLB expression in the liver of mice that were fed *ad libitum* and subjected to ZT. ^**^P < 0.01, peak *vs*. nadir, one-way ANOVA followed by Bonferroni’s *posthoc* test, *n* = 5. RT-qPCR (**C**) and Western blot (**D**) analyses of POLB expression in the liver of mice subjected to 16-h fasting or 16-h fasting, followed by 20-h refeeding. ^**^P < 0.01 *vs*^.^ fasted 16 h group, one-way ANOVA followed by Bonferroni’s *posthoc* test, *n* = 5. RT-qPCR (**E**) and Western blot (**F**) analyses of POLB expression in the liver of mice subjected to time-restricted feeding. The fold changes were calculated by normalizing the raw data to ZT0 NF group. ^**^P < 0.01 NF12 *vs*. NF0^, ##^*P* < 0.01 DF12 *vs*. DF0, one-way ANOVA followed by Bonferroni’s *posthoc* test, *n* = 5. NF night feeding, DF day feeding.

Given the liver is the largest metabolic organ with an active DNA damage/repair cycle during a day, we firstly examined the 24-h expression rhythmicity of POLB in the mouse liver. As shown in Fig. 1A, the mRNA expression of hepatic *Polb* exhibited a diurnal oscillation pattern, which reached to its nadir at ZT1 and gradually increased to its peak at ZT21. In contrast, the oscillation pattern of POLB protein expression peaked at ZT13 (Fig. 1B and Table S1). As food intake is a crucial Zeitgeber for the entrainment of circadian clocks in peripheral tissues, we also examined hepatic POLB expression in response to food signals by using mice subjected to fasting/refeeding cycles and time-restricted feeding. We found that the mRNA expression levels of *Polb* were unaltered in these mice, whereas POLB protein expression was significantly decreased in the liver of refed mice (Fig. 1C, D). Furthermore, the oscillation of POLB protein expression was reversed by restricted feeding, suggesting that this protein is controlled by both food and clock signals (Fig. 1E, F). In addition, the expression of other core BER components, such as FEN1, APEX1 and X-ray repair cross-complementing protein 1 (XRCC1), was insensitive to these signals and was kept stable in our settings (Fig. S1A–C and Tables S1, 2).

### The dysfunction of POLB impairs hepatic circadian homeostasis in vivo and in vitro

**Fig. 2:**
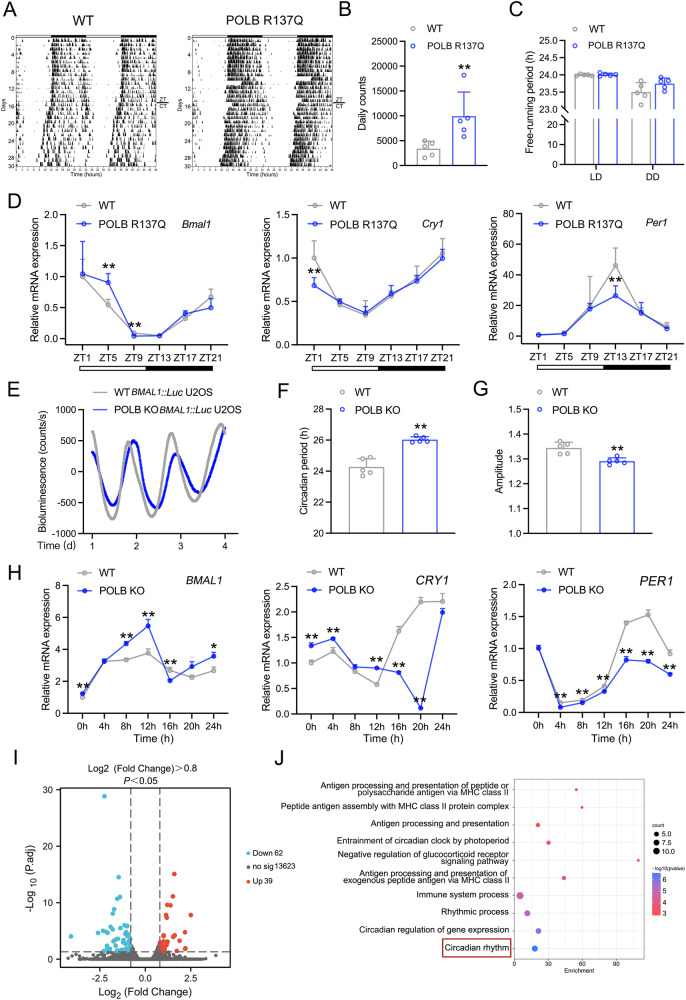
The dysfunction of POLB impairs hepatic circadian homeostasis in vivo and in vitro. **A** Representative actograms of wheel-running activities of WT and POLB^*R137Q*^ mice. **B** The calculation of mouse daily counts in Fig. 2A. **C** The calculation of mouse free-running period Fig. 2A. ^**^P < 0.01 *vs*. WT group, one-way ANOVA followed by Bonferroni’s *posthoc* test, *n* = 5. **D** RT-qPCR analyses of clock genes expression in the liver of WT and POLB^*R137Q*^ mice. ^**^P < 0.01 *vs*^.^ WT group, one-way ANOVA followed by Bonferroni’s *posthoc* test, *n* = 5. **E** Representative luminescence traces of WT and *POLB* KO *BMAL1*::*Luc* U2OS cells stimulated with dexamethasone for 2 h. The periods (**F**) and amplitudes (**G**) of circadian transcriptional activities of *BMAL1* promoter in WT and *POLB* KO *BMAL1*::*Luc* U2OS cells stimulated with dexamethasone for 2 h. ^**^P < 0.01 *vs*. WT group, one-way ANOVA followed by Bonferroni’s *posthoc* test, *n* = 5. **H** RT-qPCR analyses of time-course expression of clock genes in WT and *POLB* KO HepG2 cells similarly treated in Fig. 2E. ^**^P < 0.01 *vs*. WT group, one-way ANOVA followed by Bonferroni’s *posthoc* test, *n* = 3. **I** Volcano plot showing differentially expressed genes in the liver of POLB^*R137Q*^ mice. **J** GO analysis of biological pathways. *n* = 3. All values are presented as the mean ± SD.

To evaluate the potential role of POLB in maintaining the circadian homeostasis, we first examined the wheel-running activity of POLB^*R137Q*^ mice. As shown in Fig. 2A–C, the POLB^*R137Q*^ mice were hyperactive accompanied with a slightly prolonged period under constant dark compared to the WT mice. In contrast, the food intake and body weight of POLB^*R137Q*^ and WT mice were similar (Fig. S2A). At the molecular level, the circadian oscillation patterns of mRNA expression of core clock genes, including *Bmal1*, *Cry1* and *Per1*, were dampened in the livers of POLB^*R137Q*^ mice (Fig. 2D and Table S3). In detail, Meta2d analysis indicated that the amplitudes of *Cry1* and *Per1* oscillation were decreased in these mutant mice (Table S4). As expected, the rhythmicity of these clock genes in the suprachiasmatic nucleus remained unaltered (Fig. S2B and Tables S3, 4). Additionally, the protein expression rhythmicity of POLB was modestly altered in the liver of POLB^*R137Q*^ mice, as evidenced by a minor advanced phase (~0.35 h advance) and decreased amplitude (~29.38% reduction) (Fig. S2C and Table S5). To confirm the effects of POLB on clock homeostasis, we challenged human WT and *POLB* KO *BMAL1*::U2OS cells (the validation of *POLB* KO efficiency was presented in Fig. S2D) with a dexamethasone shock, followed by real-time bioluminescence analysis. We found that *POLB* deficiency led to a significant prolonged period (24.27 ± 0.54 h *vs*. 26.03 ± 0.18 h, *P* = 0.0001), while the amplitude of activity rhythms was slightly decreased (Fig. 2E–G). To achieve a more physiological relevance, we repeated above experiments in HepG2 cells with *POLB* deficiency (*POLB* KO efficiency was presented in Fig. S3A). As shown in Fig. 2H, dexamethasone shock induced a robust oscillation of all examined clock genes, including *BMAL1*, *CRY1* and *PER1*. However, *POLB* deficiency disrupted the circadian patterns of these genes (Fig. 2H and Table S6). In detail, the circadian amplitudes of *BMAL1* and *CRY1* were increased, whereas *PER1* was decreased (Table S7).

To globally identify downstream molecular events in POLB-orchestrated transcriptional network, we performed the transcriptome analysis and found that mRNA expression levels of total 101 genes were altered in the livers of POLB^*R137Q*^ mice (Fig. 2I). Notably, these genes were mostly enriched into the circadian clock-associated pathway (Fig. 2J).

### Dysfunction of POLB reduces HCC progression in a circadian manner in vivo

**Fig. 3:**
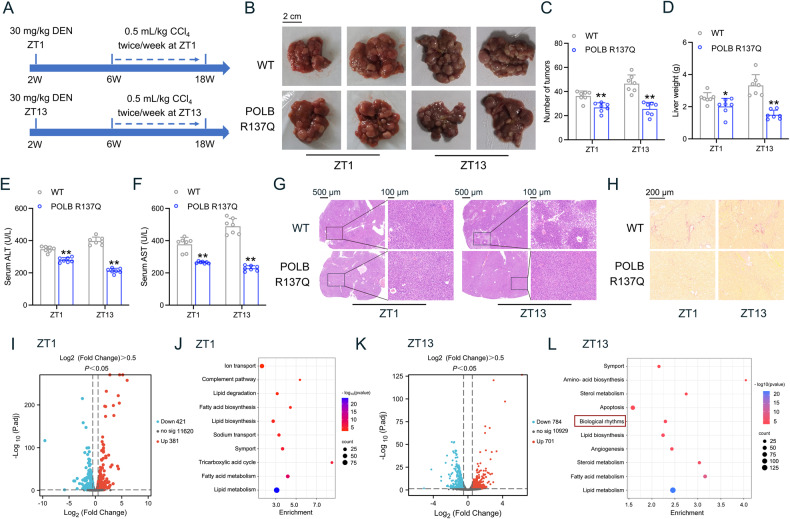
The dysfunction of POLB reduced HCC progression in a circadian manner in vivo. **A** A schematic diagram illustrating the HCC mouse model. POLB^*R137Q*^ mice and WT littermates were subjected to DEN plus CCl_4_ treatment at ZT1 and ZT13 respectively. **B** Liver images. **C** Tumor numbers. **D** Liver weight. **E** Serum ALT. **F** Serum AST. ^*^P < 0.05 and ^**^P < 0.01 *vs*^.^ WT group, one-way ANOVA followed by Bonferroni’s *posthoc* test, *n* = 7. All values are presented as the mean ± SD. **G** Representative images of H&E staining for liver sections. Scale bar = 500 μm (left), 100 μm (right). **H** Representative images of Sirius red staining for liver sections. Scale bar = 200 μm. **I** Volcano plot showing differentially expressed genes in the liver tumor tissues of POLB^*R137Q*^ mice at ZT1. **J** GO analysis of biological pathways at ZT1. **K** Volcano plot showing differentially expressed genes in the liver tumor tissues of POLB^*R137Q*^ mice at ZT13. **L** GO analysis of biological pathways at ZT13. *n* = 2. ZT Zeitgeber time.

Since both POLB-driven BER process and circadian clock are extensively involved in cancer development, we next evaluated the impacts of POLB on HCC progression from a chronobiological view. To induce HCC, POLB^*R137Q*^ mice and WT littermates were subjected to diethylnitrosamine (DEN) plus CCl_4_ treatment at ZT1 and ZT13, respectively (Fig. 3A). As shown in Fig. 3B, C, the number of liver tumors was reduced in POLB^*R137Q*^ mice compared to WT mice. Consistently, the liver weight and the serum alanine aminotransferase (ALT)/aspartate transaminase (AST) levels were significantly decreased in mutant mice (Fig. 3D–F and Table S8). Histologically, H&E staining analysis showed that the tumors in the WT group were composed of densely packed cells, whereas the POLB^*R137Q*^ group showed reduced tumor cell density and blurred cell borders (Fig. 3G). Sirius red analysis indicated a marked alleviation in hepatic portal fibrosis in POLB^*R137Q*^ mice (Fig. 3H). Notably, the retardation of HCC progression by POLB^*R137Q*^ mutation was more apparent when the carcinogen was injected at ZT13. For instance, the liver weight was decreased by 21.54% in POLB^*R137Q*^ mice at ZT1, while it was decreased to 54.87% at ZT13. Similarly, the reduction percentage of serological AST levels was 53.26% at ZT13 compared to 29.82% at ZT1. To identify downstream molecular events globally, high-throughput RNA-seq analysis was performed in these HCC samples. Volcano analysis indicated that mRNA expression levels of total 802 genes were altered in the liver tumor samples of POLB^*R137Q*^ mice at ZT1 (Fig. 3I). GO enrichment analysis revealed that these genes were clustered into the lipid metabolism pathway (Fig. 3J). Meanwhile, a cluster of 1485 genes was significantly changed at ZT13 (Fig. 3K). Importantly, the biological rhythmic pathway was also enriched in the liver of POLB^*R137Q*^ mice carrying HCC at ZT13 (Fig. 3L). It is worthwhile to point out that POLB is a DNA polymerase functionally located in the nucleus, we thus analyzed the cellular location of these downstream genes and found that only clock-associated genes were mostly enriched in the nucleus (Table S9), suggesting the modulation of circadian clock is a critical pathophysiological output of POLB, therefore the POLB^*R137Q*^ mutant alleviated HCC progression in a circadian-dependent manner, especially at ZT13.

To confirm the roles of POLB in tumorigenesis in vitro, we evaluated cell behaviors in both *POLB* KO and WT HepG2 cells. As shown in Fig. S3B, *POLB* deficiency significantly decreased the cell proliferation rate. These results were confirmed by using an EdU incorporation assay (Fig. S3C, D). As is known, cell proliferation is tightly related to the cell cycle re-entry. Coincided with above findings, *POLB* deficiency arrested more cells at the G0–G1 phase and blocked the S phase entry (Fig. S3E). Moreover, a subcutaneous xenograft tumor model established by *POLB* KO HepG2 cells exhibited reduced tumor volumes and weights (Fig. S3F–H). Therefore, POLB is a bona fide oncogene involved in the pathogenesis of liver cancer.

### POLB activates *Per1* mRNA expression by inducing epigenetic CpG demethylation

**Fig. 4:**
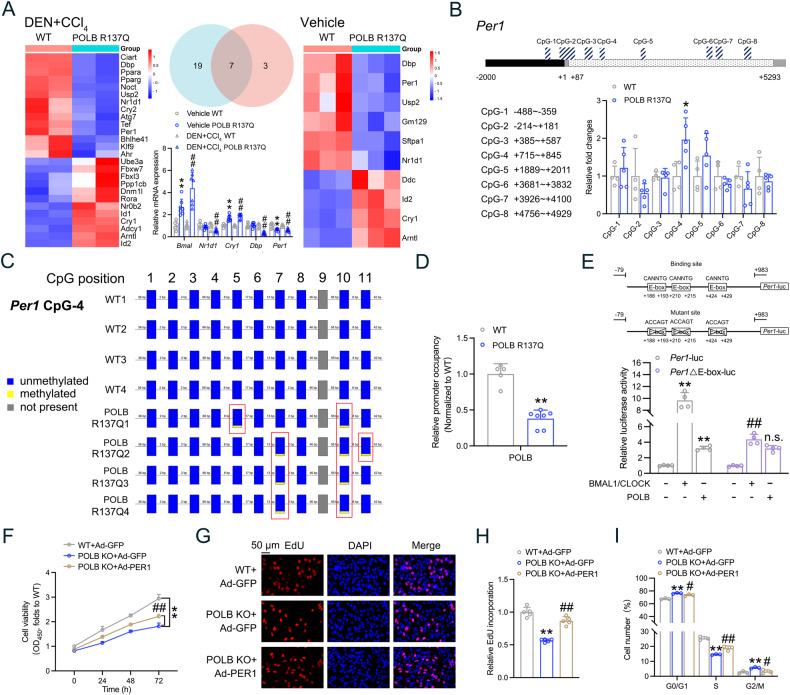
POLB activates *Per1* mRNA expression by inducing epigenetic CpG demethylation. **A** Heatmap of genes enriched in biological rhythm pathway from the liver of POLB^*R137Q*^ HCC (left) and non-tumor mice (right) at ZT13. Middle: Venn analysis and RT-qPCR validation for mRNA expression levels of five clustered genes. ^**^P < 0.01 *vs*. Vehicle WT group, ^##^*P* < 0.01 *vs*^.^ DEN + CCl_4_ group, one-way ANOVA followed by Bonferroni’s *posthoc* test, *n* = 5~7. **B** Up: a schematic outline of the eight CpG islands in the promoter (−2000bp~+1 bp) and 5′UTR of mouse *Per1*. Down: MedIP analysis of methylation level of each CpG island in the liver of WT and POLB^*R137Q*^ mice. ^*^P < 0.05 *vs*. WT group, one-way ANOVA followed by Bonferroni’s *posthoc* test, *n* = 5~7. **C** BSP analysis of the methylation level of 4th CpG island in the liver of WT and POLB^*R137Q*^ mice. **D** ChIP assays with indicated antibodies in the liver of WT and POLB^*R137Q*^ mice. The enrichment of POLB was quantified by RT-qPCR analysis. ^**^P < 0.01 *vs*. WT group, one-way ANOVA followed by Bonferroni’s *posthoc* test, *n* = 5~7. **E** Reporter gene assays in mouse PHs transfected with indicated plasmids. ^**^P < 0.01 *vs*. the basal levels, ^##^*P* < 0.01 *vs*^.^ co-transfection of *Per1*-luc and BMAL1 + CLOCK group, one-way ANOVA followed by Bonferroni’s *posthoc* test, *n* = 4. *POLB* KO HepG2 cells were infected with adenoviruses encoding GFP (Ad-GFP) or Per1 (Ad-Per1) for 48 h. **F** CCK-8 assays were performed 24-h later after adenoviruses infected to detected cell proliferation. ^**^P < 0.01 *vs*. WT + Ad-GFP group, ^##^*P* < 0.01 *vs. POLB* KO + Ad-GFP group, one-way ANOVA followed by Bonferroni’s *posthoc* test, *n* = 6. **G** EdU incorporation assay used to measure cell proliferation. **H** Quantification of the EdU incorporation assay presented in Fig. 4G. ^**^P < 0.01 *vs*. WT + Ad-GFP group, ^##^*P* < 0.01 *vs*. POLB KO + Ad-GFP group, one-way ANOVA followed by Bonferroni’s *posthoc* test, *n* = 5. **I** Assessment of the cell cycle progression by flow cytometry. ^**^P < 0.01 *vs*. WT + Ad-GFP group, ^##^*P* < 0.01 *vs. POLB* KO + Ad-GFP group, one-way ANOVA followed by Bonferroni’s *posthoc* test, *n* = 3. All values are presented as the mean ± SD.

Since POLB is rhythmically expressed in the liver and it is reciprocally required for clock homeostasis, we performed Venn analysis to investigate downstream circadian effectors. Seven genes were identified to be consistently regulated in the liver of POLB^*R137Q*^ HCC and non-tumor mice. An independent RT-qPCR analysis confirmed the mRNA expression patterns of *Bmal1*, *Cry1* and *Per1* (Fig. 4A). It should be noted that POLB is involved in the DNA demethylation process to activate genetic transcription [26], therefore we hypothesize that POLB^*R137Q*^ mutant may reasonably repress downstream gene expression. Given that *Per1* was the only downregulated gene, it may serve as the authentic target in the POLB-driven cascade flux. In this sense, we analyzed the CpG islands on the proximal promoter and 5′UTR, which were closed to the transcriptional start site of *Per1*. As shown in Fig. 4B, eight CpG islands were predicted by Ensembl and MethPrimer. MedIP analysis revealed that the 4th CpG island presented on the 5′UTR of *Per1* was hypermethylated in the liver tissues of POLB^*R137Q*^ mice at ZT13 (Fig. 4B). Such a hypermethylation was further confirmed by bisulfite sequencing PCR (BSP) analysis (Fig. 4C). Coincidence with these results, ChIP assays indicated that POLB was located near the 4th CpG island on the 5′UTR of *Per1*, while POLB^*R137Q*^ significantly reduced such recruitment (Fig. 4D).

In addition to the epigenetic modification of CpG islands, it has been documented that an E-box motif residing in the 5′UTR also regulates the gene transcriptional activity [27]. We found that three E-box motifs were present in this region (−79~+ 983 bp). To clarify the role of POLB in the regulation of the demethylation of the *Per1* 5′UTR, we constructed a *Per1* luciferase reporter containing both the 4th CpG island and the mutated E-box motifs. Reporter gene assays demonstrated that as expected, BMAL1 and CLOCK synergistically activated *Per1* transcription. Similarly, overexpression of POLB also promoted *Per1* transcription even when the E-boxes were mutated, suggesting an E-box independent regulation in *Per1* gene transcription by POLB (Fig. 4E). In addition, the presence of 8-oxoG in CpG sequences significantly hinders the methylation of neighboring cytosine residues [28, 29]. Consistent with the activity of POLB, liver tumors from POLB^*R137Q*^ mice exhibited an enrichment of 8-oxoG DNA damage. This accumulation was particularly pronounced when the carcinogen was administered at ZT1 (Fig. S4). To dissect the role of Per1 in mediating POLB’s function, we recapitulated Per1 expression in *POLB* KO HepG2 cells. As shown in Fig. 4F–H, overexpression of PER1 partially relieved the inhibition of cell proliferation triggered by *POLB* deficiency. Similar results were observed in cell cycle analysis (Fig. 4I). All these findings suggest that POLB/PER1 axis positively contributes to the HCC progression. To further confirm this hypothesis, we knocked out *PER1* in HepG2 cells (*PER1* KO efficiency was presented in Fig. S5A) and found that *PER1* deficiency led to a reduced cell proliferation rate (Fig. S5B–D). In vivo xenograft tumor assays revealed that *PER1* KO reduced tumor volumes and weights in HCC-bearing mice (Fig. S5E–G).

### The expression of POLB is increased in HCC specimens

**Fig. 5:**
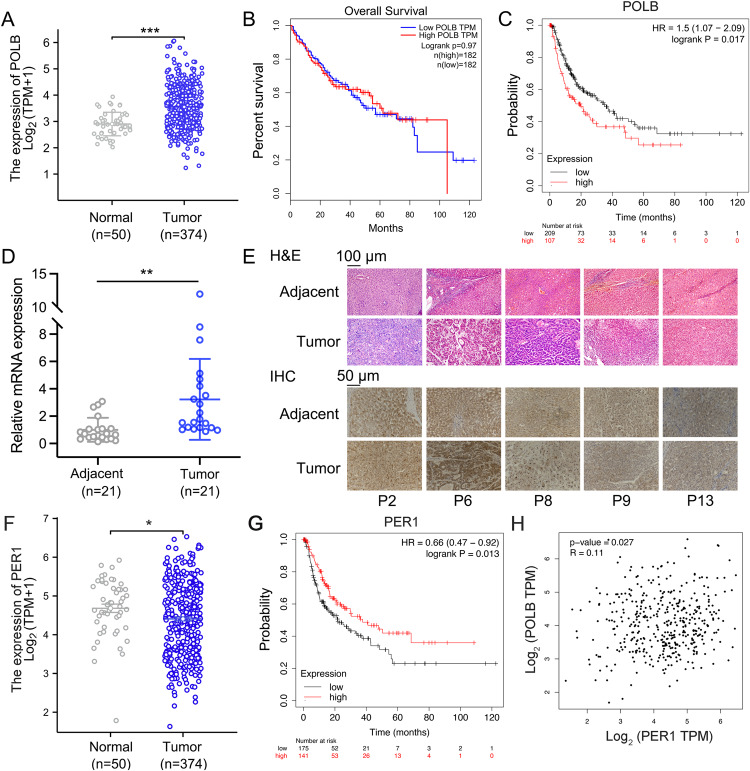
The expression of POLB is increased in HCC specimens. **A** The expression of POLB in HCC tissues (*n* = 372) and adjacent normal liver tissue (*n* = 50) from Cancer Genome Atlas database. ^***^P < 0.001 *vs*. normal group, one-way ANOVA followed by Bonferroni’s *posthoc* test. **B** Kaplan–Meier overall survival curves for all 364 patients with HCC stratified by high and low expression of POLB. **C** Kaplan–Meier survival analysis of POLB in patients with HCC using KMplot (http://kmplot.com). **D** RT-qPCR analysis of *POLB* mRNA expression from HCC tumor and adjacent non-tumor tissues. ^**^P < 0.01 *vs*. adjacent group, one-way ANOVA followed by Bonferroni’s *posthoc* test, *n* = 21. **E** Representative H&E staining and IHC analysis of POLB expression in HCC tumor and adjacent non-tumor tissues. **F** The expression of PER1 in HCC tissues (*n* = 372) and adjacent normal liver tissue (*n* = 50) from Cancer Genome Atlas database. **G** Kaplan–Meier survival analysis of PER1 in patients with HCC using KMplot (http://kmplot.com). **H** The correlation of *POLB* and *PER1* mRNA levels in the both LIHC and normal samples.

The TCGA analysis revealed that mRNA expression levels of *POLB* were increased in liver hepatocellular carcinoma (LIHC) samples (374 cases) compared to their adjacent normal tissue samples (50 cases) (Fig. 5A). Although low expression levels of *POLB* did not affected the overall survival of HCC patients, they were positively correlated with disease-free survival, as evidenced using the public database KM plotter (Fig. 5B, C). To validate the database results, we examined *POLB* mRNA expression in a cohort of 21 paired HCC and matched adjacent normal tissue samples. Patients’ detailed clinical and pathological information were presented in Table S10. Our results revealed that the mRNA and protein expression of POLB was significantly increased in HCC tissues compared to their adjacent normal controls (Fig. 5D, E). In addition, we performed a TCGA analysis for the mRNA expression of PER1 in LIHC patient samples. As shown in Fig. 5F, the mRNA expression levels of *PER1* were modestly decreased in LIHC samples (374 cases), when compared to their adjacent normal tissue samples (50 cases). Meanwhile, high expression levels of PER1 were positively correlated with disease-free survival (Fig. 5G). These results seem to be contradictory to that of POLB in LIHC patient samples. Such an inconsistency may be due to significant differences in the mRNA expression of the PER1 gene in both LIHC and normal samples. However, the mRNA levels of *POLB* and *PER1* were positively correlated (*R* = 0.11 and *P* = 0.027) in the both LIHC and normal samples (Fig. 5H), indicating the positive correlations of these two genes in clinic.

### POLB is post-translationally regulated by CALR

**Fig. 6:**
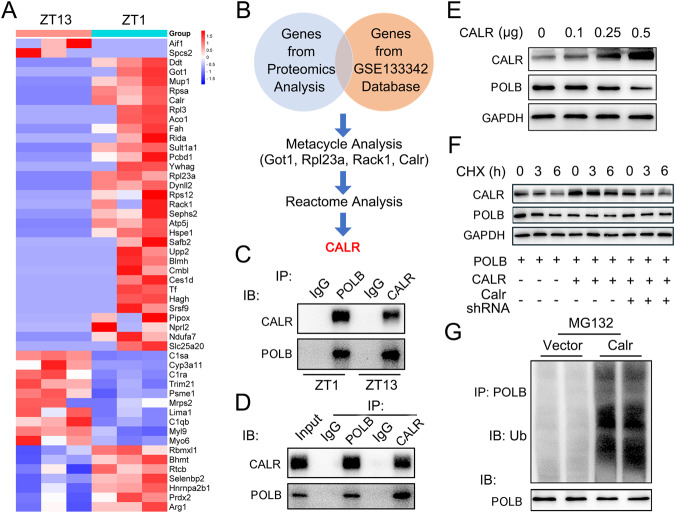
POLB is post-translationally regulated by CALR. **A** Heatmap of genes derived from label-free proteomics analyses. **B** A schematic model of the bioinformatics screening for Calr. **C** Co-IP assays in the liver lysates from ZT1 and ZT13 mice. Immunoblots of precipitated proteins were performed by using indicated antibodies. *n* = 3. **D** Co-IP assays in mouse PHs. Immunoblots of precipitated proteins were performed by using indicated antibodies. *n* = 3. **E** Western blot analyses of POLB and CALR expression in mouse PHs transfected with indicated amounts of plasmids encoding mouse Calr. *n* = 3. **F** The CALR-induced degradation of POLB protein were assessed by CHX chase experiments. *n* = 3. **G** Ubiquitination of POLB protein were detected by Western blot. All values are presented as the mean ± SD.

Although the POLB/PER1 axis has been successfully established and its function in HCC progression has been clarified in the above studies, how the rhythmicity of POLB is achieved remains unknown. To answer this question, we performed Co-immunoprecipitation (Co-IP, IP: anti-POLB) and label-free proteomics analyses by using mouse liver samples obtained at ZT1 and ZT13. As shown in Fig. 6A, a total of 50 proteins was changed according to the circadian time point. However, we are still unable to accurately identify the key molecules that regulate the rhythmic expression of the POLB protein. To address this issue, we next clustered them with our previous RNA-seq database (GSE133342), which is a collection of hepatic gene profiles in response to the constant darkness and fasting/refeeding cycles [30] (Fig. 6B). Our objective of this clustering analysis is to utilize our own transcriptome dataset to identify prominent genes that respond to external signals, including clock and food cues. This allowed us to swiftly identify genes that possess clock robustness at the transcriptional level and subsequently refine our focus to uncover potential upstream genes involved in regulating the protein stability of POLB. Note that the rhythmic robustness of a gene in the circadian clock is defined as the expression pattern of the gene, which exhibits significant fluctuations in response to the daily changing external signals, including the light/dark and fasting/refeeding cycles. Hence, using Metacycle analysis, we identified four genes (*Got1*, *Rpl23a*, *Rack1* and *Calr*) as potential candidates with a robust rhythmic expression pattern (Table S11). Among which, only *Got1* and *Calr* were found to be responsive to the refeeding signals (Table S12). Reactome analysis (https://reactome.org) further narrowed down these two factors and indicated that only *Calr* was involved in the posttranslational protein modification pathway. In addition, we found that the mRNA and protein expression levels of hepatic CALR exhibited robust diurnal fluctuations, and that CALR was sensitive to both fasting/refeeding and time-restricted feeding signals and exhibited opposite expression patterns compared to the POLB protein (Fig. S6A–C and Table S13). Therefore, we selected CALR as the target mediator of the synergy between food and peripheral clock signals on the POLB protein expression. To confirm this, we performed an independent Co-IP analysis, which showed that CALR abundantly bound with POLB in the mouse liver and PHs. Note that their binding was decreased at ZT13, when compared to ZT1 (Fig. 6C, D and Fig. S6D, E). Moreover, overexpression of CALR in mouse PHs led to a dose-dependent decrease in the POLB protein expression (Fig. 6E and Fig. S6F). To further investigate the mechanism by which CALR regulates POLB protein levels, we performed a cycloheximide (CHX) chase experiment, which showed that overexpression of CALR accelerated the protein degradation of POLB, while knockdown of *Calr* using shRNA blocked such a degradation (Fig. 6F). Accordingly, the ubiquitination of POLB was found to be dramatically increased in mouse primary hepatocytes (PHs) transfected with CALR-expressing plasmids (Fig. 6G).

In conclusion, CALR is finely orchestrated by both food and peripheral clock signals, and consequently triggers protein ubiquitination of POLB, culminating in the circadian fluctuation of POLB protein.

## Discussion

**Fig. 7:**
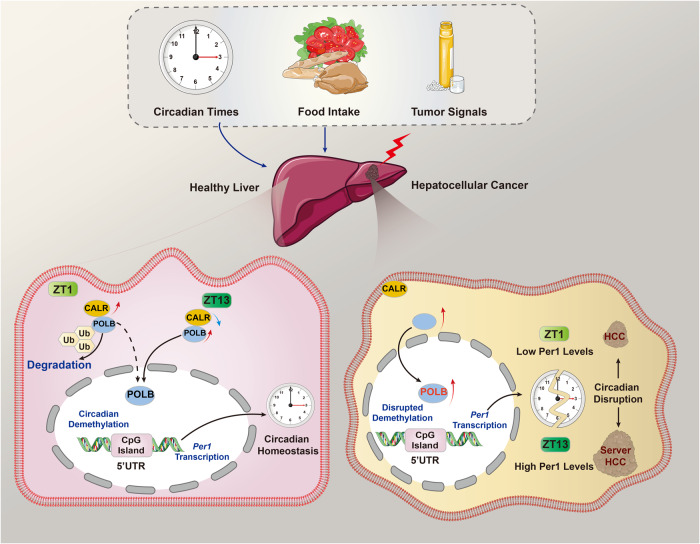
A schematic model illustrating DNA POLB connects tumorigenicity with the circadian clock in liver cancer through the epigenetic demethylation of *Per1*.

The circadian clock is implicated in various cellular functions, including DNA repair, through a transcription-translation feedback loop. A decade ago, researchers already found that the expression levels of XPA, one of the key components in the nucleotide excision repair system, exhibited robust diurnal oscillation, providing the first piece of evidence that DNA repair is controlled by the circadian clock [31]. However, the circadian oscillation of BER is still unclear. In our present study, we analyzed the expression pattern of core components in the BER system throughout a day, and found that while the expression levels of XRCC1, FEN1, and APEX1 were roughly stable, POLB possessed a precise circadian expression pattern and was responsive to food signals, especially at its protein level. Moreover, the pathophysiological activities of both POLB^*R137Q*^ mutant mice and *POLB* KO HepG2 cells exhibited a dampened circadian pattern, and more importantly, POLB dysfunction retarded HCC progression in a circadian-dependent manner. Coincided with these findings, POLB expression was upregulated in human HCC samples and was positively correlated with poor prognosis. At the molecular level, POLB was involved in the demethylation of the 4th CpG island on the 5′UTR of core clock gene *Per1* and activated its transcription, leading to enhanced tumorigenicity. Besides, considering the nadir and peak times of hepatic POLB protein expression at ZT1 and ZT13, we later performed proteomics and bioinformatics clustering analysis with our previous database and screened out CALR as an important regulator that triggered POLB protein ubiquitination in response to both clock and food signals. Hence, our findings provide important insights into the molecular mechanism underlying the synergy between clock and food signals on the POLB-driven BER system and new clock-dependent carcinogenetic effects of POLB (Fig. 7). Chronobiological modulation of POLB may help to promote precise interventions for HCC.

Epidemiologic studies indicate that circadian dysfunction among night-shift workers and individuals suffering from sleep dyspnea is a significant risk factor for the development of cancers, including HCC [21, 32, 33]. Consistently, chronic circadian misalignment is sufficient to induce spontaneous hepatocarcinogenesis in mice [21]. Furthermore, mice exposed to the UV carcinogen at 4:00 a.m. exhibit a decreased latency and an approximately 5-fold increased multiplicity of skin cancer compared to mice exposed to UV at 4:00 p.m. [34]. All these phenomena indicates that the circadian clock plays a vital role in carcinogenesis. However, studies are lacking to identify direct molecule targets that relay the clock signals to tumorigenicity. To filling in this missing piece of the puzzle, we established a mouse HCC model by injection of DEN and CCl_4_ according to the peak and nadir time points of POLB protein expression, and revealed a time-dependent on the chemical-induced hepatocarcinogenesis at ZT1 and ZT13. In particular, HCC mice established by chemical injection at ZT13 exhibited more severe tumorigenesis phenotypes, when compared to HCC mice established at ZT1. More importantly, POLB dysfunction with R137Q mutant significantly alleviated the HCC progression, and its anti-tumor effects were more profound at ZT13, indicating the circadian clock is potentially involved in relaying the oncogenic signal of POLB. Indeed, the transcriptome data from the liver of normal and tumor-baring mice were clustered, and the circadian rhythm was identified as the most responsive pathway to POLB signals. These results implied that disrupted clocks contributed to the circadian discrepancy of anti-tumor properties observed in the POLB deficiency background on HCC progression (Fig. 3). Currently, the roles of POLB in the tumorigenesis are still in debate. On one hand, the expression levels of POLB have been found to be elevated in certain tumors, including lung cancer, breast cancer, and colon cancer [26, 35]. On the other hand, POLB variants with low catalytic activity are also linked to an increased risk of cellular transformation and cancer susceptibility [7, 36, 37]. Overexpression of POLB decreases the metastasis and invasion of lung and breast cancers [26]. Of note, POLB is a core component in BER system, and inhibition of BER pathway has been proven to decrease the prostate cancer cell proliferation and is a potential therapeutic strategy for prostate cancer treatment [38]. Consistently, we found that genetic mutation of POLB (reduced enzymatic activity) reduced the liver tumorigenesis, and in vitro studies further confirmed the anti-tumor properties of POLB dysfunction (Fig. 3 and Fig. S3). Therefore, at least in our study, POLB’s role in HCC progression is positive and it serves as a promising integrator linking the circadian clock signals to liver cancer development. Of note, our results may conflict with the traditional understanding of a proficient BER safeguarding against mutation accumulation and tumorigenesis. It should be noted that if the POLB was not mutated, the POLB’s preservation of genomic integrity may remain consistent in either normal cells or tumor cells [39]. Hence, under normal physiological conditions, POLB aids in the identification of damaged DNA fragments by normal cells, thereby initiating the DNA repair process to avert genomic instability. In tumor cells, POLB might additionally contribute to the preservation of the tumor cell genome integrity. In our study, as liver cancer was induced using chemical mutagens, the mutagenesis process led to the initiation of liver cell carcinogenesis. Consequently, POLB within the cells may have already recognized and adapted to the mutant genome of the tumor, regarding it as the correct genomic sequence. Hence, even at ZT13, the peak time of POLB expression, it is conceivable that POLB may contribute to the stabilization of the genome induced by mutagens, thereby promoting tumor development. In addition, PER1, as one of the critical genes involved in tumor cell resistance to apoptosis, may also partially receive and amplify oncogenic signals from POLB.

Accumulating evidence have demonstrated that epigenetic modification, such as DNA methylation, play an important role in cancer progression [40, 41]. Moreover, DNA methylation on the promoter of core circadian genes controls their mRNA expression levels, thus maintaining the circadian clock homeostasis [42]. Inhibition of DNA methylation can modify chromatin structure and affect gene expression, influencing cell fate decision [43]. Notably, the BER system is essential for the AP-site repair generated by TDG, potentially involving in the TET1-TDG-BER-mediated DNA demethylation [44]. Active global DNA demethylation was *de facto* found to be associated with the activity of BER, while BER proteins participate in the process of DNA demethylation [45, 46]. POLB, as a core component of BER system, has been shown to exert demethylation abilities closely associated with lung and breast cancer metastasis [26]. In addition to the classic DNA methylation region existed on the gene promoter, the 5′UTR is another important region for DNA methylation modification [47, 48]. Our results revealed that POLB is involved in the demethylation of the *Per1* 5′UTR, further activating its mRNA expression. Functionally, overexpression of PER1 partially abolished the POLB deficiency-induced cell proliferation and G0/G1 phase cell cycle arrest of HepG2 (Fig. 4). Since the amount of endogenous POLB protein was rhythmically enriched, the demethylation function of POLB was different at each time point, producing a time discrepancy of chemical-induced HCC progression. For instance, when the hepatic POLB protein level reached its peak time at ZT13, the abundant POLB executed its demethylation function on the 5′UTR of *Per1*, further aggravating HCC progression. More importantly, such a demethylation-triggered *Per1* transcription was persistent even when the E-box were mutated, indicating that POLB-mediated 5′UTR demethylation is a non-classical pathway that activates *Per1* transcription independent of the BMAL1/CLOCK heterodimer. This non-classical pathway may explain the inconsistent results of cancer chronochemotherapy based on the circadian pattern of key clock genes, including BMAL1 and CLOCK.

PER1 expression is diminished in a variety of malignancies, such as prostate cancer, colon cancer, cholangiocarcinoma, gastric cancer, and non-small cell lung cancer. PER1 is associated with unfavorable prognosis in these patients, whereas upregulation of PER1 confers anti-tumor characteristics against the aforementioned cancers [49–52]. However, the drug target role of PER1 in cancer remains a subject of debate. The precise role of PER1 in the apoptosis regulation has yet to be completely elucidated, since different studies have reported conflicting findings regarding its pro- and an anti-apoptotic function [53–55]. For example, it has been reported that PER1 enhances the sensitivity of cancer cells to ionizing radiation-induced apoptosis by inhibiting p21-mediated cell cycle arrest through induction of c-Myc [53]. In contrast, an anti-apoptotic role of PER1 has been observed in various human cancer cell lines [54, 55]. More importantly, given PER1 is a central clock gene that controls circadian homeostasis, it is imperative to examine the true role of PER1 in tumor progression under circadian conditions. In our study, we found that the incidence of tumors was higher when carcinogen was administered at the peak of PER1 expression, specifically at ZT13 (Fig. 3). One potential explanation for this could be that the peak expression of PER1-driven anti-apoptotic genes in tumor cells also occurs at ZT13, resulting in increased tumor survival and development at this specific time point.

Protein posttranslational modification is a critical process that determines protein function, localization, stability, and interaction with other molecules [56]. In the present study, a significant circadian misalignment of POLB was observed at its transcriptional and translational levels. Hence, we speculated that such an uncoupling is potentially caused by protein posttranslational ubiquitination, which is orchestrated by the reciprocal synergy between food and clock signals. Taking advantages of proteomics and bioinformatics analyses, we identified that CALR is responsible for triggering POLB protein ubiquitination (Fig. 6). CALR is a calcium-binding endoplasmic reticulum protein that plays multiple roles in the government of protein folding, and facilitation of their secretion and insertion in the plasma membrane, thus contributing to the maintenance of cellular homeostasis when unfolded proteins accumulate within the endoplasmic reticulum [57–59]. Given that excessive unfolded protein accumulation disrupts the circadian oscillation in the mouse liver and inhibits fasting-induced gluconeogenesis [60, 61], we hypothesized that CALR is potentially regulated by clock and nutritional signals. Indeed, the expression levels of hepatic CALR exhibited robust circadian rhythmicity and were actively responsive to fasting/refeeding cycles (Fig S6, Tables S11–13). Hence, CALR may serve as an integrator linking the peripheral clock and food signals to regulate POLB in a circadian-dependent manner. On the other hand, recent studies have unveiled the promising role of CALR as a protein in cancer treatment. A large number of research evidence has indicated the relevance of CALR to multiple types of tumors, including hematologic malignancies and solid tumors [62–64], implying CALR has the potential to function as a therapeutic target and a biomarker for specific types of tumors. In HCC, the mRNA expression of *CALR* is elevated compared to normal tissue, while higher mean levels of anti-CALR IgG antibiodies are detected in the sera of HCC patients compared to healthy individuals [65]. At the molecular level, CALR plays a crucial role in activating phosphorylation of the PI3K/Akt pathway, which in turn sustaining the malignant behavior of HCC cells [66]. Additionally, this CALR is controlled by Stat3-eIF2α axis, which is essential for CALR translocation [67, 68]. In our study, CALR exhibited an anti-phase expression pattern compared to that of POLB in healthy liver cells, contradictory to the facts that CALR actually is an oncogene during HCC progression and both CALR and POLB are consistently upregulated in human HCC condition (Fig. 5A) [65]. Such a paradox can be explained by the different cellular location and functions of CALR in the normal and tumor settings. It has been revealed that cancer cells succumb to immunogenic cell death and then expose intracellular CALR to the surface of their cell membranes [69, 70]. This process facilitates the uptake of cell corpses by specialized phagocytes and ultimately triggers the onset of anti-cancer immunity [69]. Such a translocation of CALR and its dissociation from POLB exhibit some degree of heterogeneity that is linked to cell type and initiating trigger, resulting in varying expression and functions of Calr in different types of normal and cancer settings.

In conclusion, our study focused on the role of POLB in the regulating the circadian clock and HCC progression. Our findings shed some light on the mechanism by which POLB maintains circadian homeostasis, and triggers the HCC progression in a circadian-dependent manner by mediating the demethylation on the 5′UTR of *Per1*. Because POLB is clinically upregulated in human HCC samples and positively correlated with patient poor prognosis, targeting POLB from a chronobiological perspective could be an attractive strategy for the precise intervention in HCC.

## Materials and methods

### Animals

All animal procedures in this investigation conform to the Guide for the Care and Use of Laboratory Animals published by the US National Institutes of Health (NIH publication No. 85-23, revised 1996) and the approved regulations set by the Laboratory Animal Care Committee at China Pharmaceutical University (Permit number SYXK-2021-0011). All mice were maintained in a 12 h light/dark cycle and in a temperature- and humidity-controlled environment. Zeitgeber time zero (ZT0) referred to lights on. To establish the circadian mouse model, as well as the starvation and refeeding mouse model and time-restricted feeding mouse model, 8-week-old male mice in the C57BL6/J background were used, as previously reported [30]. To investigate the role of POLB in HCC progression, we constructed HCC mouse models using POLB^*R137Q*^ male mice and their wild-type (WT) littermate in the 129S1 background. We selected the POLB^*R137Q*^ variant as it had only 30% primer extension activity compared to the WT enzyme, whereas global *Polb* knockout (KO) causes mouse embryonic death [11]. These mice were generously provided by Professor Zhigang Guo’s lab (Nanjing Normal University, Nanjing, Jiangsu, China). To create the HCC mouse model, 14-day-old male mice were given a single injection (*i.p*.) of 30 mg/kg DEN at either ZT1 or ZT13. 6-week later, these mice received CCl_4_ (0.5 mL/kg) injection (*i.p*.) twice a week at either ZT1 or ZT13 [71]. To investigate the effect of POLB on xenograft tumor growth in vivo, *POLB*-deficient (POLB KO) HepG2 cells (5 × 10^6^ cells per mouse) were injected subcutaneously into the right flanks of male BALB/C nude mice (GenePharmatech, Nanjing, Jiangsu, China). Tumor volumes (V = length × width × width/2) were measured every three days.

### RT-qPCR and Western blot analyses

Total RNA was isolated using Trizol reagent (Invitrogen, Carlsbad, CA, USA), reverse transcribed, and analyzed by qPCR using SYBR Green (Vazyme, Nanjing, Jiangsu, China) and the LightCycler^®^ 480 System (Roche, Basal, Switzerland). The primers for human *β-ACTIN* and mouse *36B4* were included for normalization when indicated. A complete list of PCR primers is shown in Table S14. For protein expression analysis, liver tissues were homogenized, and the cells were lysed in RIPA buffer. Equal amounts of protein were loaded and separated by 10% SDS-PAGE, then transferred onto polyvinylidene difluoride membranes (Millipore, Bedford, MA, USA). The membranes were incubated overnight with appropriate primary antibodies and bound antibodies were then visualized using HRP-conjugated secondary antibodies. A quantitative analysis was performed by using AlphaEaseFC software (AlphaInnotech, San Leando, CA, USA). The antibodies against POLB (Cat. No. ab26343; 1:1000 dilution) and CALR (Cat. No. ab2907; 1:1000 dilution) were purchased from Abcam (Cambridge, MA, USA). Antibodies against XRCC1 (Cat. No. 21468-1-AP; 1:1000 dilution), APEX1 (Cat. No. 10203-1-AP; 1:1000 dilution), FEN1 (Cat. No. 14768-1-AP; 1:1000 dilution), and GAPDH (Cat. No. 60004-1-AP; 1:5000 dilution) were purchased from Proteintech (Chicago, IL, USA). Uncropped images are provided in Original data.

### Cell culture

Mouse primary hepatocytes were isolated from mice by using the collagenase IV (Gibco, Grand Island, NY, USA) perfusion method as described previously and cultured in a humidified atmosphere containing 5% CO_2_ at 37 °C [72]. Both *POLB* KO, *PER1* KO HepG2 cells and *POLB* KO human *BMAL1*::*Luc* U2OS cells were established using a CRISPR/Cas9 system according to the standard protocol provided by Zhang’s lab [73]. Briefly, we designed the single guide RNA (sgRNA) using the online CRISPR Design Tool (http://crispr.mit.edu/) and cloned them into SpCas9-2A-puro vector (PX459). After confirming the cutting efficiency of the sgRNA, we transfected the SpCas9 vectors into cells. Puromycin-resistant cells were sorted and seeded onto 96-well plates for monoclonal colonization. After four weeks, cell clones derived from single cells were screened for gene expression by DNA sequencing. The following sgRNA sequences targeting human *POLB* and *PER1* were used. sgRNA for *POLB* knockout were: GAGTCTAGAGGCGCATCTCAG; sgRNA for *PER1* knockout were: GTGCTAACCTGTGAGCATGT.

### Rhythmicity of gene expression

The rhythmicity of gene expression was assessed using the “meta2d” function in the “Metacycle” R package [74]. “Metacycle” is designed to detect periodic signals and integrate the results from the ARSER, JKT-CYCLE, and Lomb–Scargle methods. In this study, genes were considered rhythmically expressed when the meta2d_pvalue was <0.05.

### Wheel-running activity

Ten-week-old mice were housed in standard mouse cages equipped with infrared sensors to detect locomotor activity. After being synchronized to a 12-h light/dark cycle for at least 7 days, the mice were transferred into light/dark for 15 days, followed by constant dark for another 15 days. Wheel revolutions were recorded in 6-min intervals by using ClockLab system (Actimetrics, Wilmette, IL, USA). The circadian period and phase shift in DD were determined using ClockLab analysis software (Actimetrics, Wilmette, IL, USA) [75, 76].

### Real-time monitoring assay

For the real-time monitoring assay, human *BMAL1*::*Luc* U2OS cells (a gift from Zhang Eric Erquan [77], National Institute of Biological Sciences, Beijing, China) were plated onto 35-mm dishes (6 × 10^5^ cells/dish) were cultured at 37 °C and 5% CO_2_–95% air in high-glucose DMEM supplemented with 10% FBS for 5 days, and then the culture medium was then changed into serum-free DMEM for an additional 24 h, followed by a 2-h dexamethasone shock. To achieve real-time circadian variation, the medium was replaced with recording medium and raw data were analyzed as previously described [78]. The period length and amplitude were calculated from the raw data derived from 24 to 120 h after synchronization using MATLAB software (9.0 Version R2016a, MathWorks Inc., MA, USA) [30].

### Dexamethasone shock

To perform the dexamethasone shock, HepG2 cells were cultured with DMEM plus 100 nM dexamethasone for 2 h. Following this, the HepG2 cells were washed once with PBS and incubated with serum-free DMEM (Time point = 0 h). Cell samples were collected at 4-h intervals, and total RNA was extracted and processed for RT-qPCR analysis.

### High-throughput RNA sequencing

Liver samples were collected to screen out candidate genes that mediate the role of POLB in regulating the liver clock and HCC. Total RNA was isolated from the liver samples for construction of RNA-seq libraries. The RNA libraries were sequenced using a HiSeq 3000 sequencing platform (Illumina Company, USA) by RiboBio (Guangzhou, Guangdong, China). For additional statistics, Fragments Per Kilobase of transcript per Million mapped reads (FPKM) were calculated. All reads were mapped to the mouse genome (GRCm38/mm10). The DAVID tool was used for pathway enrichment and gene cluster analysis (https://david.ncifcrf.gov/).

### Serological analysis

Blood samples were collected in non-heparinized tubes and centrifuged at 4000 rpm for 10 min at 4 °C. The serum levels of ALT and AST were determined spectrophotometrically using commercial kits (Jiancheng Institute of Biotechnology, Nanjing, Jiangsu, China).

### H&E, Sirius red staining and Immunofluorescence

Liver samples from both human and mouse HCC were isolated, fixed in 4% paraformaldehyde solution for 24 h in situ, processed for paraffin embedding, and cut into 4 µm transverse sections. H&E and Sirius red staining were performed on the sections. The slides were scanned using a Pannoramic Flash 250 scanner (Perkin Elmer, Waltham, MA, USA) and viewed using the Pannoramic viewer software program (3D Histech, Waltham, MA, USA). Immunofluorescence was conducted to detect the oxidative DNA damage using mouse anti-8-oxoG (sc130914, Santa Cruz, Dallas, TX, USA). After washing, the slides were probed with secondary antibodies conjugated to Alexa Fluor 594 anti-mouse immunoglobulin G (IgG; Cat. No. 33212ES60, 1:200 dilution, YEASEN, Shanghai, China) at room temperature. The sections were captured with a Nikon fluorescence microscope (ECLIPSE, Ts2R-FL, Tokyo, Japan).

### Proliferation assay

To analyze cell proliferation, CCK-8 and EdU assays were performed. For the CCK-8 assay, cells were seeded in 96-well plates at a density of 5 × 10^3^ cells per well. Subsequently, 10 μL CCK-8 reagent (Beyotime Biotechnology, Nantong, Jiangsu, China) was added to each well and incubated for 2 h. Cell proliferation was assessed by measuring the absorbance at 450 nm using a microplate reader. For EdU incorporation, HepG2 cells were seeded in 12-well plates at a density of 5 × 10^4^ cells per well for the indicated times. Then, the cells were incubated with 10 μM EdU (RiboBio, Guangzhou, Guangdong, China) for 2 h. Afterward, the cells were fixed with 4% paraformaldehyde and were double stained with DAPI (Sigma–Aldrich, St. Louis, MO, USA). Signals were visualized using a fluorescence microscope, and the average ratios between EdU-positive (red) and total DAPI-stained nuclei (blue) were calculated for statistical analyses.

### Flow cytometry

To analyze the cell cycle of HepG2 cells, the following steps were performed. The cells were fixed with 70% ethanol overnight, washed twice with PBS, and stained with 500 μL of propidium iodide (PI) (50 μg/mL PI in the sample buffer containing 0.1% Triton X-100, 0.1 mmol/L EDTA, and 100 μg/ml RNase A). After 1 h, the fluorescence of the PI-DNA complex in each nucleus was measured using a FACSCalibur instrument (Becton-Dickinson, Sunnyvale, CA, USA). The rates of the G_0_/G_1_, S, and G_2_/M phases of the cell cycle were determined using the Modfit LT software.

### CpG island analysis

The CpG sites in the promoter (−2000bp~+1 bp) and 5′UTR of mouse *Per1* were predicted by Ensembl (http://www.ensembl.org/index.html) and MethPrimer (http://www.urogene.org/cgi-bin/methprimer/methprimer.cgi). DNA methylation were detected by MedIP and BSP. For MedIP, 10 μg liver genomic DNA was sonicated and then heat denatured. 5% of the DNA was reserved as the input, while the remaining portion was divided equally. To each portion, 2 μg of IgG or 5-mC antibody was added and incubated at 4 °C overnight. Protein G beads were added to capture the complexes, and the eluted DNA was subsequently used for PCR analysis. The primer sequences were presented in Table S1. For BSP analysis, 2 μg of genomic DNA was denatured using 0.3 M NaOH for 10 min at 37 °C. After adding hydroquinone and sodium bisulfate, the samples were incubated at 50 °C for 16 h. The modified DNA was then purified and amplified by PCR. The PCR products were further purified and cloned into the pTG19-T cloning vector (Generay, Shanghai, China). At least ten positive clones from each product were selected for sequencing.

### ChIP assay

ChIP assays were performed in mouse livers essentially as described previously [72]. Briefly, chromatin lysates were prepared, pre-cleared with Protein-A/G agarose beads, and immunoprecipitated with antibodies against POLB or normal mouse IgG (Cat. No. SC-2025, Santa Cruz, Dallas, TX, USA) in the presence of BSA and salmon sperm DNA. The beads were extensively washed before reverse cross-linking. The DNA was purified using a PCR purification kit (Qiagen, Valencia, CA, USA) and subsequently analyzed by PCR using primers flanking the 4th CpG island on the 5′UTR of *Per1*. The primer sequences were the same to the primers used in MedIP analysis.

### Transfection and reporter gene assays

The E-box binding sites that were proximal to the key CpG island on the 5′UTR of mouse *Per1* were mutated and synthesized by Tsingke (Beijing, China). All transient transfections were conducted using Lipofectamine 3000 (Invitrogen, Carlsbad, CA, USA) according to the manufacturer’s instructions. For Calr knockdown, shRNA targeting mouse *Calr* were designed, validated, and synthesized by GenePharma (Shanghai, China). Detailed shRNA sequences were: 5′- GCAAGAATGTGCTGATCAATTCAAGAGATTGATCAGCACATTCTTGCTTTTTT-3′ for Calr; 5′- GTTCTCCGAACGTGTCACGTTTCAAGAGAACGTGACACGTTCGGAGAACTTTTTT-3′ for scramble. For the luciferase reporter assays, 200 ng of reporter plasmids were mixed with expression constructs for either mixture mouse BMAL1 (250 ng) and CLOCK (250 ng) or POLB (500 ng) alone. Equal amounts of DNA were used for all transfection combinations by adding the appropriate vector DNA. Relative luciferase activities were determined 48 h following transfection using the Luciferase System (Promega, Madison, WI, USA). The data were representative of at least four independent experiments.

### Clinical samples from public database/TCGA database analysis

HCC samples (374 cases) and adjacent normal tissue samples (50 cases) were downloaded from the TCGA-Liver hepatocellular carcinoma (LIHC) database and POLB expression was assessed. To compare the percentage survival between different POLB expression groups, Kaplan–Meier survival curves were generated. Additionally, we also assessed the impact of POLB on survival using the public database KM plotter (https://kmplot.com).

### Human subjects and IHC analysis

HCC tissue samples and the paired adjacent non-tumor tissues used in RT-qPCR and IHC analysis were surgically dissected from patients at First Affiliated Hospital of Nanjing Medical University. The study was approved by the Institutional Ethics Committee of the First Affiliated Hospital of Nanjing Medical University. Informed consent for tissue analysis was obtained before the liver biopsy or surgery.

### Co-IP analysis

The liver tissues were homogenized, and the cells were lysed in the IP lysis buffer. After centrifugation, 40 μg of protein lysate was incubated with 20 μL of Protein-A/G agarose beads (Roche, Basal, Switzerland) and 1 μg of anti-POLB antibody. Following a 12-h incubation, the immune complexes were centrifuged and washed four times with ice-cold IP wash buffer. The immunoprecipitated protein was then analyzed by using a western blot assay.

### Label-free quantification and protein identification

To identify the candidate gene that mediates the clock-dependent ubiquitination of hepatic POLB, we conducted label-free proteomics analysis using Co-IP samples (immunoprecipitated with the anti-POLB antibody) from the livers of mice sacrificed at ZT1 and ZT13. Detailed proteomics analysis was performed according to standard protein digestion and LC–MS/MS analyses by Shanghai Applied Protein Technology Co (Shanghai, China). The resulting MS raw data for each sample were consolidated and analyzed for identification and quantitation using the MaxQuant (Version 1.6.14) software.

### Protein half-life and ubiquitination analyses

The half-life of POLB protein was determined using the cycloheximide (CHX) chase assay. Mouse primary hepatocytes (PHs) were transfected with the indicated plasmids. After 36 h, DMEM medium containing 10 μg/mL CHX (Sigma–Aldrich, St. Louis, MO, USA) were added. Cells were harvested at the specified time points and subjected to western blot analysis. The ubiquitination of POLB protein was measured by a Co-IP assay. Mouse PHs were similarly transfected with indicated plasmids for 36 h, followed by the treatment with 10 μM MG132 (Sigma–Aldrich, St. Louis, MO, USA) for 6 h. Cells were lysed and lysates were precipitated with anti-POLB antibody and pre-cleared protein-A/G PLUS-Agarose beads (Roche, Basal, Switzerland). Ubiquitinated proteins within the IP were detected by western blot using a monoclonal anti-ubiquitin antibody (Cat. No. sc-8017; 1:200 dilution, Santa Cruz, Dallas, TX, USA).

### Statistical analysis

Statistical analysis was conducted using GraphPad (version 9.5, San Diego, CA, USA). The data were presented as mean ± SD (standard deviation) for each group. One-way or two-way ANOVA followed by Bonferroni’s *posthoc* test was used to analyze time series data. A *P*-value < 0.05 was considered statistically significant. When comparing only two groups, Student’s *t*-test was used unless otherwise specified.

### Supplementary information

Figure S1

Figure S2

Figure S3

Figure S4

Figure S5

Figure S6

Supplemental Materials—Figure Legends and Tables

Original Data

Checklist

## Data Availability

The data that support this study are available within the article and its supplementary data files or available from the authors upon request.
