# Supplementary figures and images for: DNA polymerase beta connects tumorigenicity with the circadian clock in liver cancer through the epigenetic demethylation of Per1
Source: Cell Death Dis. 2024 Jan 20;15(1):78. doi: 10.1038/s41419-024-06462-7 (PMC10799862; doi:10.1038/s41419-024-06462-7)

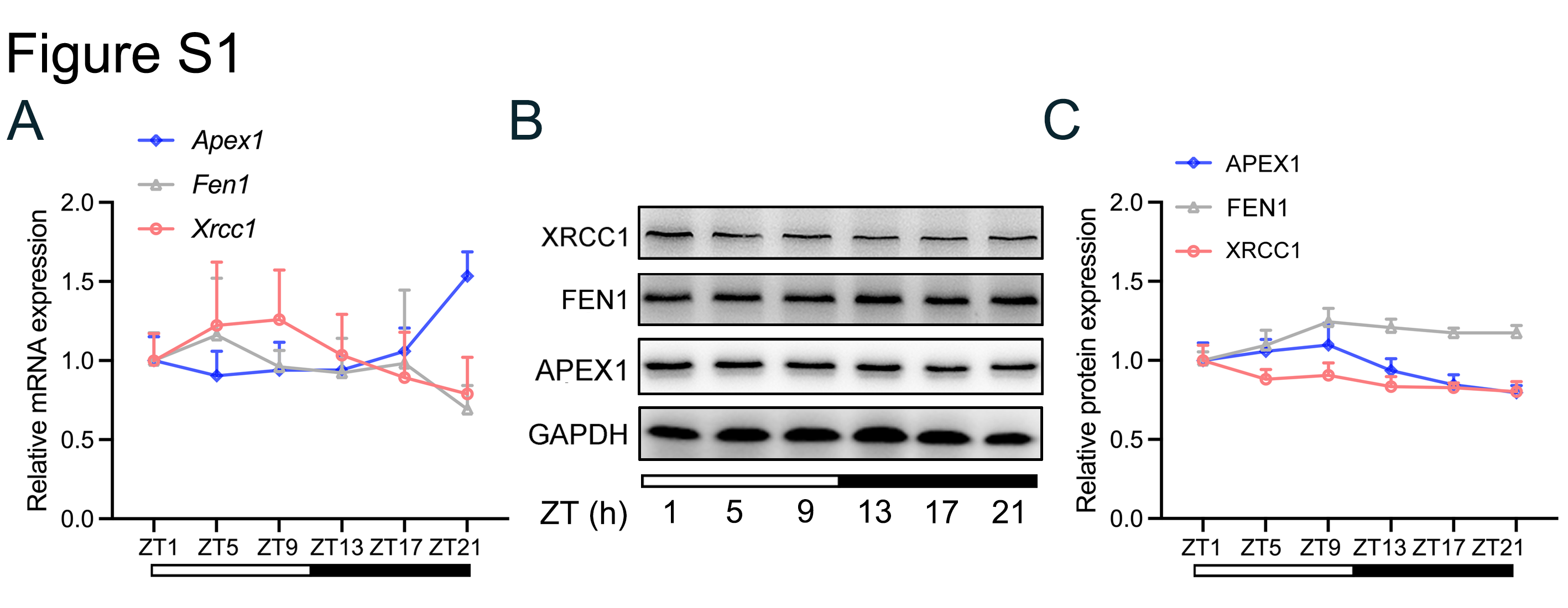

Supplement: Supplementary file 1 — Figure S1 [file 41419_2024_6462_MOESM1_ESM.tif]

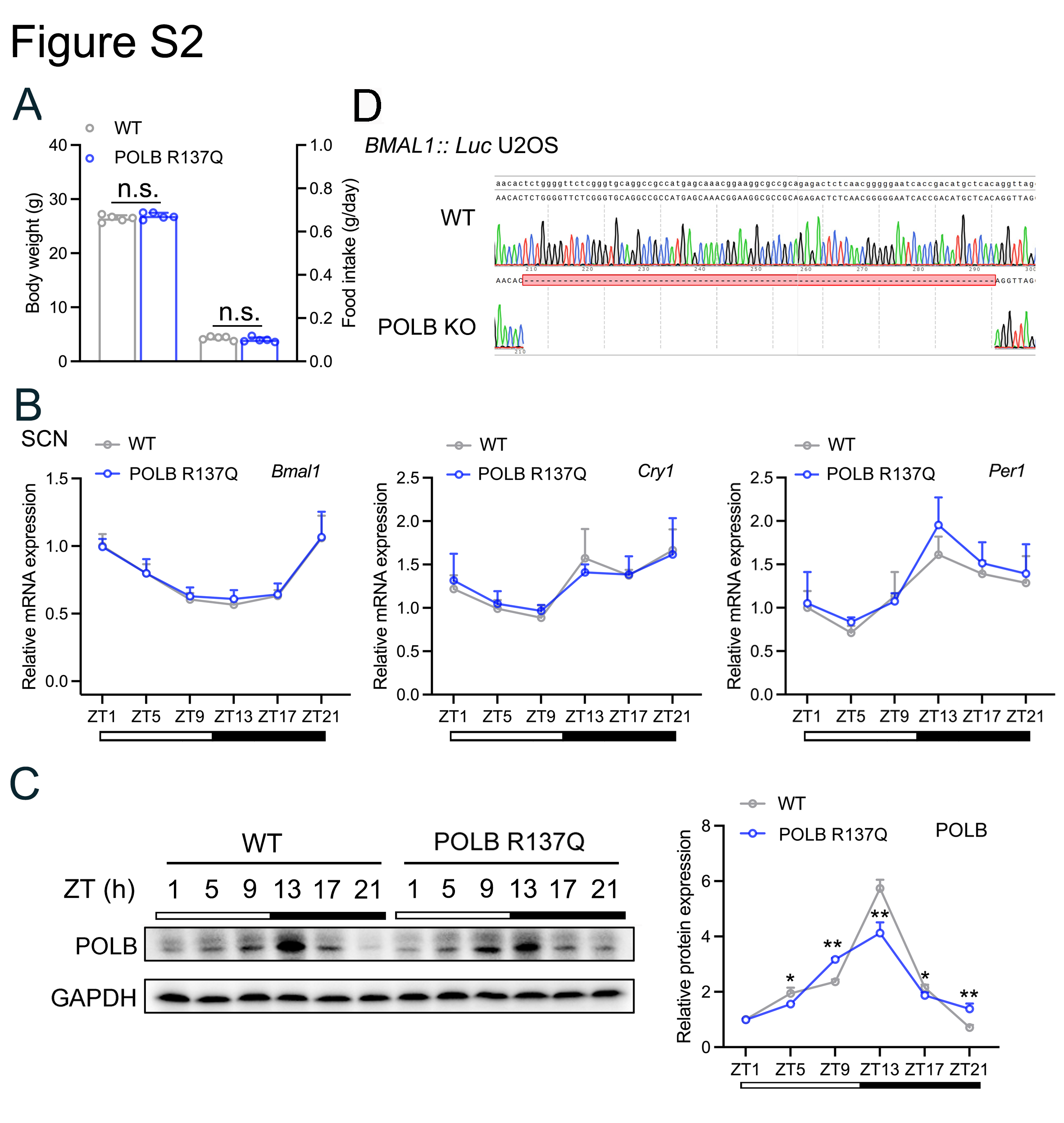

Supplement: Supplementary file 2 — Figure S2 [file 41419_2024_6462_MOESM2_ESM.tif]

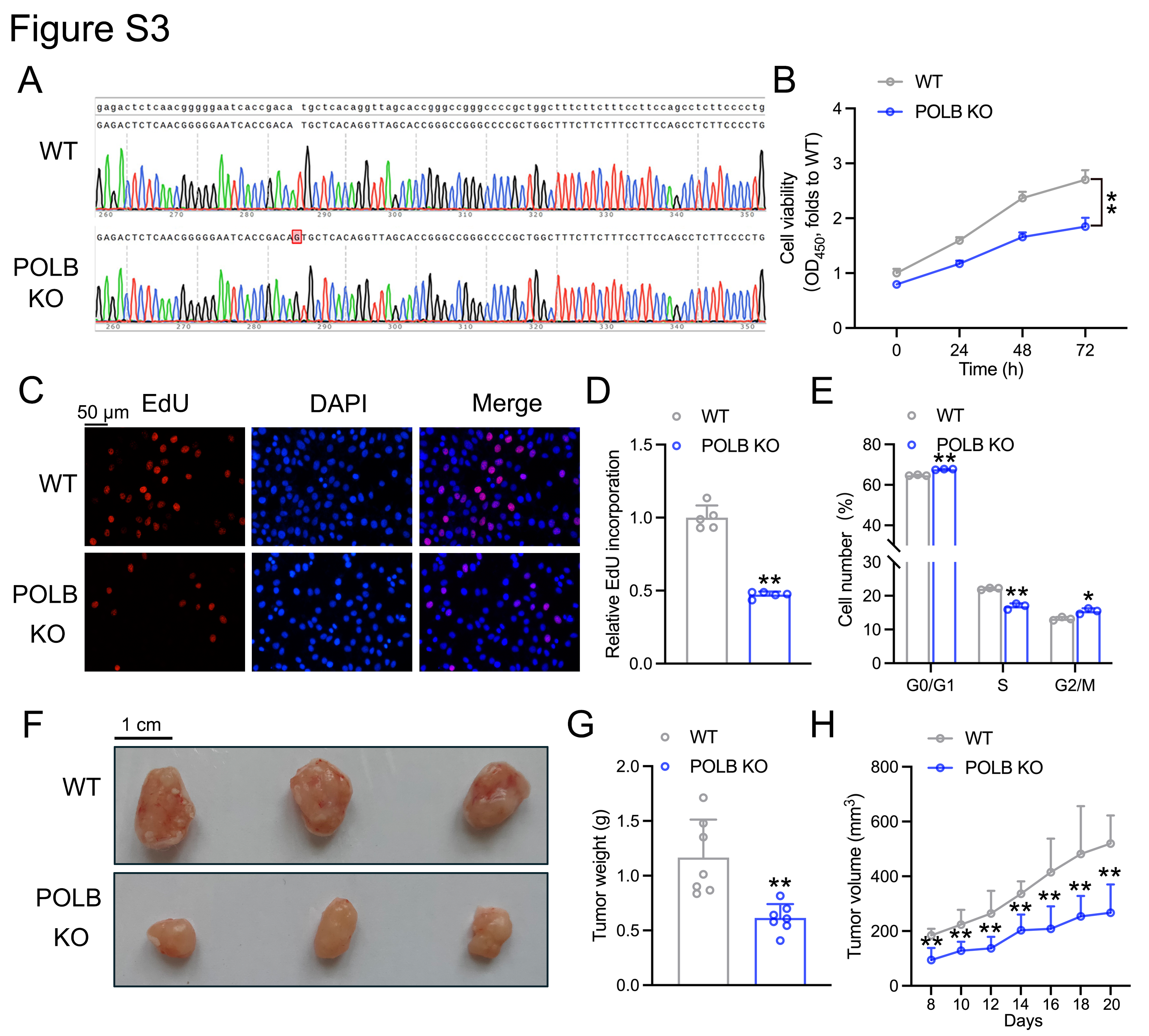

Supplement: Supplementary file 3 — Figure S3 [file 41419_2024_6462_MOESM3_ESM.tif]

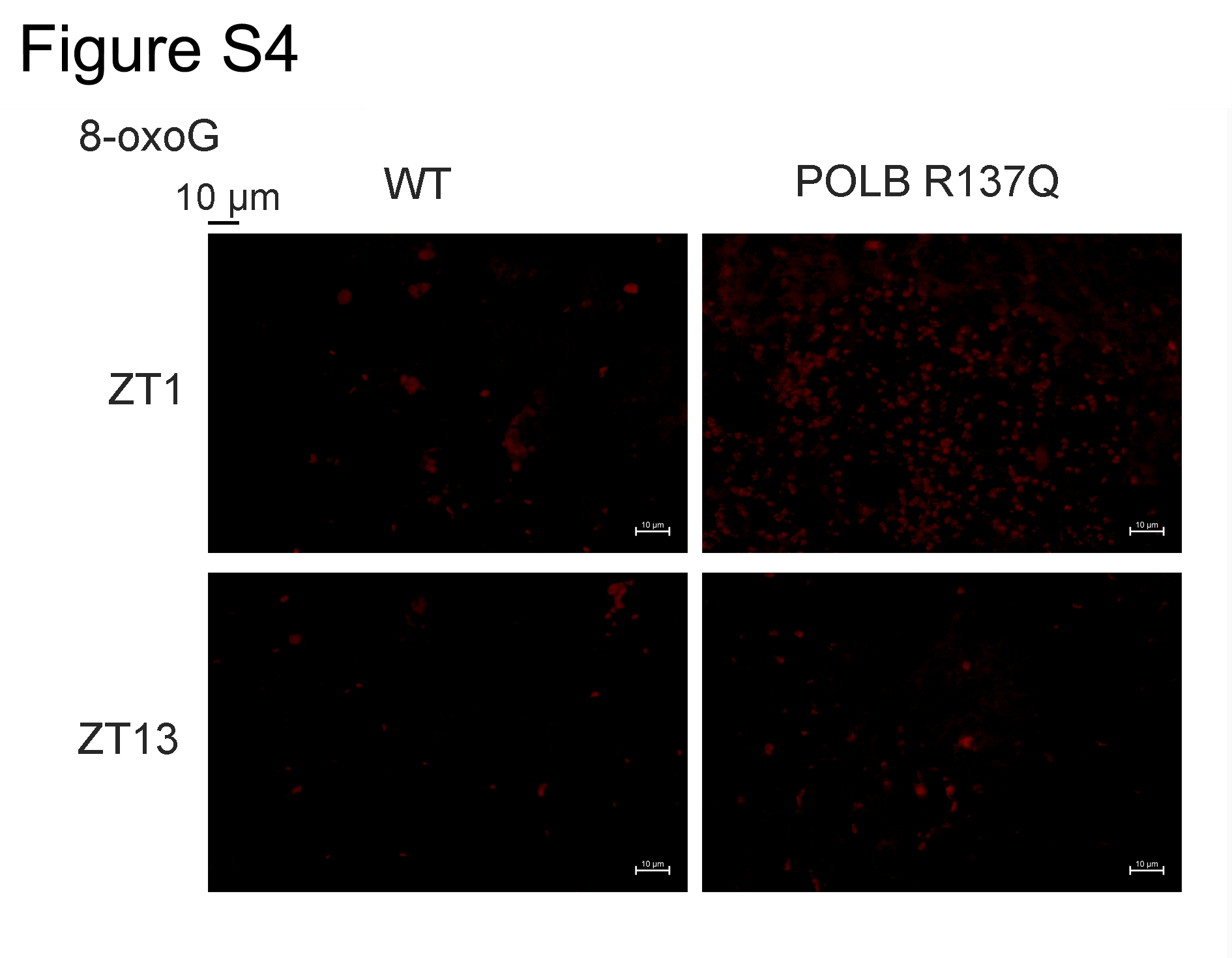

Supplement: Supplementary file 4 — Figure S4 [file 41419_2024_6462_MOESM4_ESM.tif]

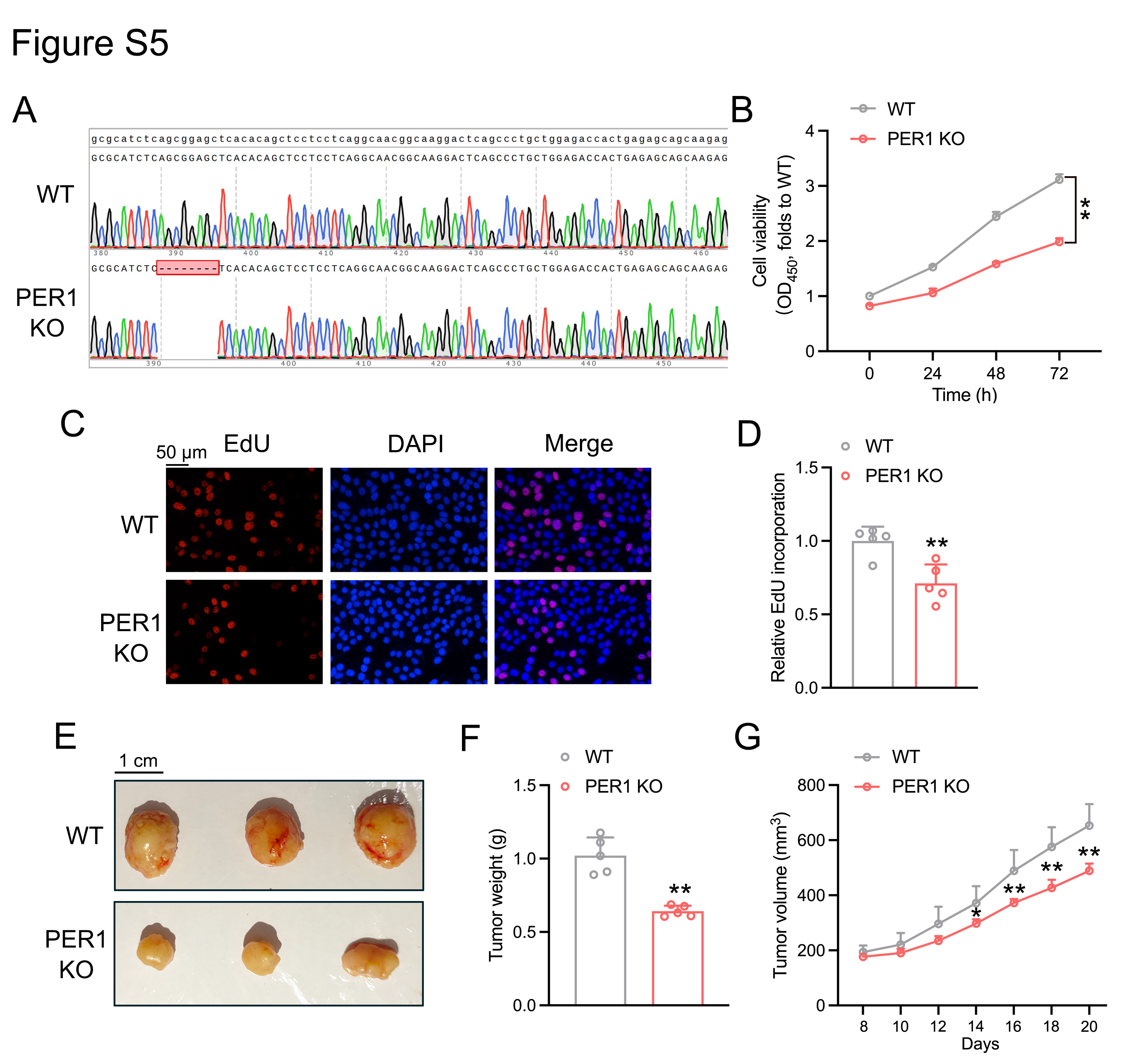

Supplement: Supplementary file 5 — Figure S5 [file 41419_2024_6462_MOESM5_ESM.tif]

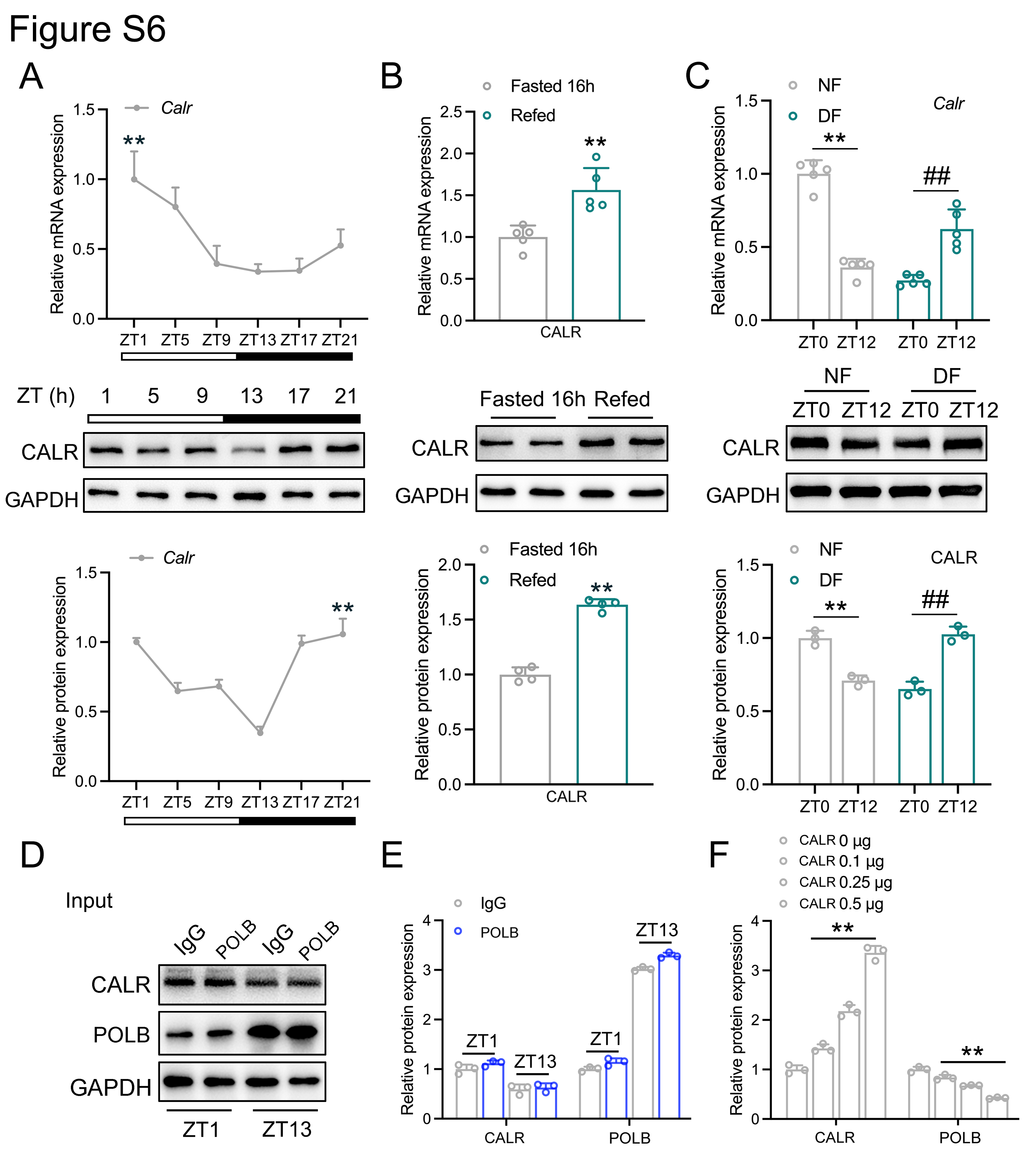

Supplement: Supplementary file 6 — Figure S6 [file 41419_2024_6462_MOESM6_ESM.tif]

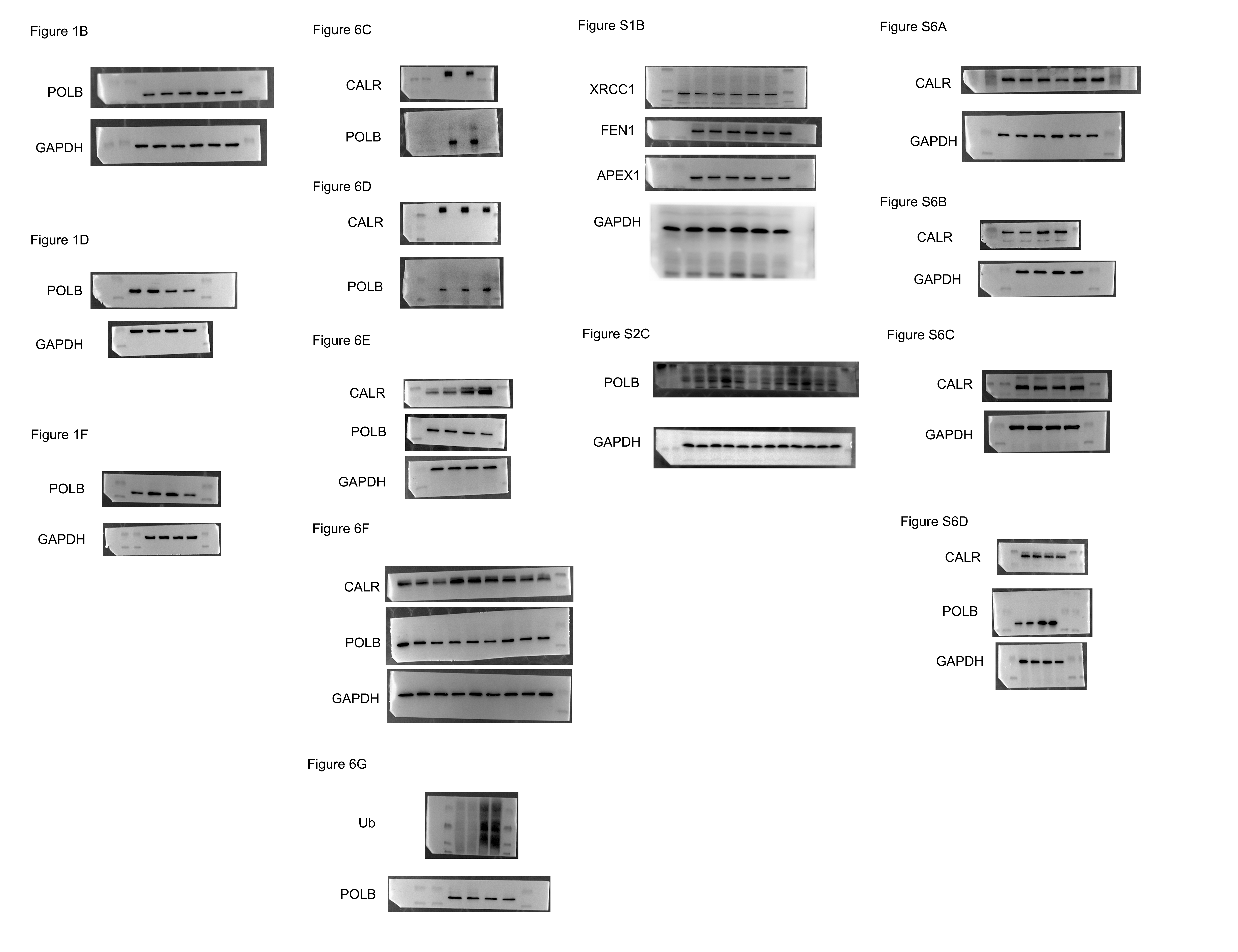

Supplement: Supplementary file 8 — Original Data [file 41419_2024_6462_MOESM8_ESM.tif]
