# Supplementary material for: DNA polymerase beta connects tumorigenicity with the circadian clock in liver cancer through the epigenetic demethylation of Per1
Source: Cell Death Dis. 2024 Jan 20;15(1):78. doi: 10.1038/s41419-024-06462-7 (PMC10799862; doi:10.1038/s41419-024-06462-7)
Supplement: Supplementary file 7 — Supplemental Materials—Figure Legends and Tables [file 41419_2024_6462_MOESM7_ESM.docx]

**Supplementary Materials**

**Supplementary Figure Legends**

**Figure S1** The rhythmic expression of BER complex components in the mouse liver. (A) RT-qPCR analyses of mRNA expression levels of BER complex components (*Apex1*, *Fen1,* *Xrcc1*) in the liver of mice subjected to ZT. n=5. (B) Western blot analyses of BER complex components (APEX1, FEN1, XRCC1) protein expression in the liver of mice subjected to ZT. n=5. (C) Quantitative analysis of BER complex components (APEX1, FEN1, XRCC1) protein expression in Figure S1B. n=5. ZT: Zeitgeber time.

**Figure S2** The dysfunction of POLB impairs hepatic circadian homeostasis. (A) Body weight and food intake of POLB*^R137Q^* mice. n=5. (B) RT-qPCR analyses of mRNA expression levels of clock genes in the SCN of WT and POLB*^R137Q^* mice. n=5. SCN, suprachiasmatic nucleus. (C) Western blot and quantitative analyses of POLB expression in the liver of POLB*^R137Q^* mice and WT littermates subjected to ZT. ^**^*P* <0.01 *vs*. WT group. One-way ANOVA followed by Bonferroni’s *posthoc* test, n=5. (D) Validation of knockout efficiency of *POLB* in *POLB* KO *Bmal1*::*Luc* U2OS cells. All values are presented as the mean ± SD.

**Figure S3** The dysfunction of *POLB* reduced HCC progression. (A) Validation of knockout efficiency of *POLB* in *POLB* KO HepG2 cells. (B) CCK-8 assays were performed to detected cell proliferation. ^**^*P* <0.01 *vs.* WT group, one-way ANOVA followed by Bonferroni’s *posthoc* test, n=6. (C) EdU incorporation assay used to measure cell proliferation. (D) Quantification of the EdU incorporation assay presented in Figure S4C. ^**^*P* <0.01 *vs.* WT group, one-way ANOVA followed by Bonferroni’s *posthoc* test, n=5. (E) Assessment of the cell cycle progression by flow cytometry. ^**^*P* <0.01 *vs.* WT group, one-way ANOVA followed by Bonferroni’s *posthoc* test, n=3. Representative tumor images (F) and tumor weights (G) at 20 days after injection with WT and *POLB* KO HepG2 cells. (H) Subcutaneous tumor volumes were measured at the indicated days. ^**^*P* <0.01 *vs.* WT group, one-way ANOVA followed by Bonferroni’s *posthoc* test, n=7. All values are presented as the mean ± SD.

**Figure S4** The dysfunction of POLB led to an elevation in the levels of 8-oxo DNA damage during the progression of HCC.

**Figure S5** *PER1* deficiency reduced HCC progression. (A) Validation of knockout efficiency of *PER1* in *PER1* KO HepG2 cells. (B) CCK-8 assays were performed to detected cell proliferation. ^**^*P* <0.01 *vs.* WT group, one-way ANOVA followed by Bonferroni’s *posthoc* test, n=6. (C) EdU incorporation assay used to measure cell proliferation. (D) Quantification of the EdU incorporation assay presented in Figure S6C. ^**^*P* <0.01 *vs.* WT group, one-way ANOVA followed by Bonferroni’s *posthoc* test, n=5. Representative tumor images (E) and tumor weights (F) at 20 days after injection with WT and *PER1* KO HepG2 cells. (G) Subcutaneous tumor volumes were measured at the indicated days. ^**^*P* <0.01 *vs.* WT group, one-way ANOVA followed by Bonferroni’s *posthoc* test, n=5. All values are presented as the mean ± SD.

**Figure S6** Hepatic CALR is regulated by clock and nutritional signals. (A) RT-qPCR and Western blot analyses of CALR expression in the liver of mice subjected to ZT. ^**^*P* <0.01, peak *vs.* nadir, one-way ANOVA followed by Bonferroni’s *posthoc* test, n=5. (B) RT-qPCR and Western blot analyses of CALR expression in the liver of mice subjected to 16-h fasting or 16-h fasting, followed by 20-h refeeding. ^**^*P* <0.01 *vs.* fasted 16 h group, one-way ANOVA followed by Bonferroni’s *posthoc* test, n=5. (C) RT-qPCR and Western blot analyses of CALR expression in the liver of mice subjected to subjected to time-restricted feeding. ^**^*P* <0.01 NF12 *vs.* NF0, ^##^*P* <0.01 DF12 *vs.* DF0, one-way ANOVA followed by Bonferroni’s *posthoc* test, n=5. (D) Western blot analysis of POLB and CALR protein expression in the liver lysates of Figure 6C. (E) Quantitative analysis of Figure S6D. n=3. (F) Quantitative analysis of POLB and CALR protein expression in Figure 6D. ^**^*P* <0.01 *vs.* 0 μg CALR group, one-way ANOVA followed by Bonferroni’s *posthoc* test, n=3.

**Supplementary Tables**

**Table S1** The metacycle analysis for the circadian expression rhythmicity of BER complex.

| **CycID** | **meta2d_**  **pvalue** | **meta2d_**  **BH.Q** | **meta2d_period** | **meta2d_**  **phase** | **meta2d_Base** | **meta2d_AMP** | **meta2d_rAMP** |
| --- | --- | --- | --- | --- | --- | --- | --- |
| **mRNA** |  |  |  |  |  |  |  |
| *Polb* | 0.0166 | 0.0277 | 24 | 14.1544 | 1.5551 | 0.1562 | 0.1005 |
| *Apex1* | 0.0008 | 0.002 | 24 | 21.0000 | 1.0628 | 0.1741 | 0.1638 |
| *Fen1* | 0.3403 | 0.3403 | 24 | 6.0209 | 0.9532 | 0.0311 | 0.0311 |
| *Xrcc1* | 0.0439 | 0.0549 | 24 | 7.2763 | 1.0331 | 0.2366 | 0.2290 |
| *Calr* | 6.5998e-10 | 3.2999e-09 | 24 | 2.0000 | 0.5677 | 0.2716 | 0.2716 |
| **Protein** |  |  |  |  |  |  |  |
| POLB | 5.1604e-06 | 2.5802e-05 | 24 | 12.6219 | 1.3826 | 0.3817 | 0.2760 |
| APEX1 | 0.0004 | 0.0011 | 24 | 8.4454 | 0.9553 | 0.0965 | 0.0965 |
| FEN1 | 0.0284 | 0.0355 | 24 | 11.3532 | 1.1494 | 0.0798 | 0.0689 |
| XRCC1 | 0.5803 | 0.5803 | 24 | 6.6577 | 0.8755 | 0.0319 | 0.0319 |
| CALR | 0.0016 | 0.0026 | 24 | 3.6840e-05 | 0.7874 | 0.2974 | 0.2974 |

**Table S2** mRNA expression levels of indicated genes annotated to the GSE133342 database.

| **Tracking_ID** | **Gene name** | **FPKM value** | | |
| --- | --- | --- | --- | --- |
|  |  | **CTL** | **Fasted 16h** | **Refed** |
| NM_009687 | *Apex1* | 1876 | 1141 | 1665 |
| NM_001271614 | *Fen1* | 621 | 123 | 611 |
| NM_009532 | *Xrcc1* | 372 | 252 | 328 |

**Table S3** F and *P* values of two-way ANOVA analysis for Figure 2D and Figure S2B.

| **Gene**  **name** | **POLB R137Q** | | **ZT** | | **POLBR137Q × ZT** | |
| --- | --- | --- | --- | --- | --- | --- |
|  | **F Values** | ***P* Values** | **F Values** | ***P* Values** | **F Values** | ***P* Values** |
| **Liver** |  |  |  |  |  |  |
| *Bmal1* | 4.085 | 0.0779 | 123.4 | <0.0001 | 7.395 | 0.0622 |
| *Cry1* | 5.893 | 0.0414 | 42.95 | <0.0001 | 3.143 | 0.0174 |
| *Per1* | 6.582 | 0.0334 | 32.57 | <0.0001 | 2.798 | 0.0293 |
| **SCN** |  |  |  |  |  |  |
| *Bmal1* | 0.1614 | 0.6984 | 41.51 | <0.0001 | 0.0984 | 0.9919 |
| *Cry1* | 0.2681 | 0.6186 | 15.54 | <0.0001 | 0.6186 | 0.6863 |
| *Per1* | 6.677 | 0.0324 | 16.91 | <0.0001 | 0.5716 | 0.7212 |

**Table S4** Metacycle analysis for the phase and amplitude of clock gene expression rhythmicity in Figure 2D and Figure S2B.

| **Liver** | | | | | | |
| --- | --- | --- | --- | --- | --- | --- |
| Gene name | meta2d_phase | | *P* value for  meta2d_phase | meta2d_AMP | | *P* value for  meta2d_AMP |
|  | WT | POLB  R137Q |  | WT | POLB R137Q |  |
| *Bmal1* | 0.8339 | 0.4987 | 0.3544 | 0.3689 | 0.5291 | 0.4610 |
|  | 0.2822 | 0.2186 |  | 0.5269 | 0.4709 |  |
|  | 0.3742 | 0.3904 |  | 0.5389 | 0.6517 |  |
|  | 0.4330 | 0.2940 |  | 0.3654 | 0.3986 |  |
|  | 0.1899 | 22.0787 |  | 0.4821 | 0.4535 |  |
| *Cry1* | 1.7529 | 22.2237 | 0.3913 | 0.2414 | 0.2296 | 0.0463 |
|  | 22.0938 | 21.3951 |  | 0.5289 | 0.2133 |  |
|  | 22.2114 | 20.9335 |  | 0.3630 | 0.2628 |  |
|  | 21.3587 | 21.5426 |  | 0.3707 | 0.2843 |  |
|  | 21.3629 | 20.8634 |  | 0.2795 | 0.1456 |  |
| *Per1* | 11.1904 | 11.9388 | 0.9688 | 24.1944 | 11.3807 | 0.0103 |
|  | 13.4849 | 12.5109 |  | 17.3068 | 15.6570 |  |
|  | 13.4746 | 13.4278 |  | 24.9874 | 16.8026 |  |
|  | 13.5823 | 12.4463 |  | 21.0002 | 8.2535 |  |
|  | 12.6201 | 13.9117 |  | 17.2053 | 14.7509 |  |
| **SCN** | | | | | | |
| Gene name | meta2d_phase | | *P* value for  meta2d_phase | meta2d_AMP | | *P* value for  meta2d_AMP |
|  | WT | POLB R137Q |  | WT | POLB R137Q |  |
| *Bmal1* | 23.0389 | 0.1979 | 0.6283 | 0.2092 | 0.3091 | 0.5456 |
|  | 0.6398 | 0.5285 |  | 0.3728 | 0.2838 |  |
|  | 23.8840 | 22.1025 |  | 0.2854 | 0.1960 |  |
|  | 1.4032 | 23.8210 |  | 0.2640 | 0.1967 |  |
|  | 0.3448 | 22.3221 |  | 0.2749 | 0.3047 |  |
| *Cry1* | 14.4869 | 21.4783 | 0.7037 | 0.4023 | 0.3669 | 0.9583 |
|  | 19.9408 | 20.5547 |  | 0.3587 | 0.4748 |  |
|  | 19.7743 | 17.5159 |  | 0.3171 | 0.5099 |  |
|  | 21.2648 | 17.6357 |  | 0.2439 | 0.1125 |  |
|  | 17.7797 | 18.8588 |  | 0.3433 | 0.1786 |  |
| *Per1* | 14.8331 | 16.6382 | 0.5544 | 0.7091 | 0.3688 | 0.6590 |
|  | 17.4397 | 15.2945 |  | 0.2497 | 0.7271 |  |
|  | 13.2567 | 12.8680 |  | 0.4024 | 0.6178 |  |
|  | 14.5542 | 13.2735 |  | 0.4052 | 0.6425 |  |
|  | 11.6131 | 17.7851 |  | 0.3823 | 0.1052 |  |

**Table S5** The metacycle analysis for the circadian expression rhythmicity of POLB in the liver of either WT or POLB*^R137Q^* mice.

| **CycID** | **meta2d_**  **pvalue** | **meta2d_**  **BH.Q** | **meta2d_period** | **meta2d_**  **phase** | **meta2d_Base** | **meta2d_AMP** | **meta2d_rAMP** |
| --- | --- | --- | --- | --- | --- | --- | --- |
| **Protein** |  |  |  |  |  |  |  |
| *WT* | 0.0768 | 0.0768 | 24 | 11.6221 | 0.6295 | 0.5724 | 0.5724 |
| POLB*^R137Q^* | 0.0052 | 0.0104 | 24 | 11.2686 | 0.5931 | 0.4042 | 0.4042 |

**Table S6** F and *P* values of two-way ANOVA analysis for Figure 2H.

| **Gene name** | ***POLB* KO** | | **Serum shock** | | ***POLB* KO ×**  **Serum shock** | |
| --- | --- | --- | --- | --- | --- | --- |
|  | **F Values** | ***P* Values** | **F Values** | ***P* Values** | **F Values** | ***P* Values** |
| *Bmal1* | 42.92 | 0.0028 | 213.7 | <0.0001 | 24.85 | <0.0001 |
| *Cry1* | 247.7 | <0.0001 | 244.6 | <0.0001 | 251.3 | <0.0001 |
| *Per1* | 416.3 | <0.0001 | 1076 | <0.0001 | 114.6 | <0.0001 |

**Table S7** Metacycle analysis for the phase and amplitude of clock gene expression rhythmicity in Figure 2H.

| **HepG2** | | | | | | |
| --- | --- | --- | --- | --- | --- | --- |
| Gene name | meta2d_phase | | *P* value for  meta2d_phase | meta2d_AMP | | *P* value for  meta2d_AMP |
|  | WT | *POLB* KO |  | WT | *POLB* KO |  |
| *BMAL1* | 10.2257 | 10.3772 | 0.1909 | 0.9771 | 1.4666 | 0.0013 |
|  | 10.9572 | 10.0381 |  | 0.9000 | 1.4183 |  |
|  | 10.4284 | 10.0368 |  | 1.0895 | 1.4535 |  |
| *CRY1* | 22.2279 | 4.7820 | <0.0001 | 0.4772 | 0.6619 | 0.0115 |
|  | 22.3877 | 3.1245 |  | 0.4813 | 0.5900 |  |
|  | 22.6015 | 5.5796 |  | 0.4950 | 0.5722 |  |
| *PER1* | 19.3576 | 19.1063 | 0.6128 | 0.7569 | 0.5777 | 0.0005 |
|  | 19.2355 | 19.4806 |  | 0.7652 | 0.5076 |  |
|  | 19.4751 | 19.2701 |  | 0.7529 | 0.5364 |  |

**Table S8** F and *P* values of two-way ANOVA analysis of relative parameters in Figure 3.

| **Parameters** | **POLB R137Q** | | **ZT** | | **POLB R137Q × ZT** | |
| --- | --- | --- | --- | --- | --- | --- |
|  | **F Values** | ***P* Values** | **F Values** | ***P* Values** | **F Values** | ***P* Values** |
| Tumor number | 53.82 | <0.0001 | 5.309 | 0.0399 | 8.775 | 0.0119 |
| Liver weight (g) | 44.53 | <0.0001 | 0.5166 | 0.4861 | 13.05 | 0.0036 |
| Serum ALT (U/L) | 564.1 | <0.0001 | 0.9347 | 0.3527 | 51.02 | <0.0001 |
| Serum AST (U/L) | 321.7 | <0.0001 | 6.608 | 0.0245 | 25.43 | 0.0003 |

**Table S9** GO analysis of cellular component of genes clustered from each biological pathway at ZT13.

| **Lipid metabolism** | | | |
| --- | --- | --- | --- |
| **Category** | **Enrichment** | ***P* value** | **Count** |
| Endoplasmic reticulum | 6.26 | 1.22E-28 | 53 |
| Microsome | 24.14 | 2.51E-25 | 24 |
| Peroxisome | 16.66 | 1.70E-11 | 13 |
| Mitochondrion | 3.19 | 1.90E-07 | 27 |
| Lipid droplet | 15.25 | 4.37E-05 | 6 |
| Membrane | 1.19 | 1.81E-02 | 77 |
| VLDL | 26.33 | 7.28E-02 | 2 |
| **Fatty acid metabolism** | | | |
| **Category** | **Enrichment** | ***P* value** | **Count** |
| Microsome | 34.65 | 6.29E-12 | 10 |
| Peroxisome | 39.73 | 3.90E-11 | 9 |
| Mitochondrion | 6.92 | 2.37E-10 | 17 |
| Endoplasmic reticulum | 6.51 | 2.65E-09 | 16 |
| Mitochondrion outer membrane | 10.47 | 3.16E-02 | 3 |
| **Steriod metabolism** | | | |
| **Category** | **Enrichment** | ***P* value** | **Count** |
| Endoplasmic reticulum | 9.42 | 4.35E-07 | 9 |
| Microsome | 44.54 | 2.68E-06 | 5 |
| Membrane | 1.65 | 1.62E-02 | 12 |
| **Angiogenesis** | | | |
| **Category** | **Enrichment** | ***P* value** | **Count** |
| Secreted | 4.62 | 3.33E-05 | 25 |
| Extracellular matrix | 8.42 | 4.54E-02 | 25 |
| **Lipid biogenesis** | | | |
| **Category** | **Enrichment** | ***P* value** | **Count** |
| Endoplasmic reticulum | 7.89 | 1.58E-09 | 14 |
| Lipid droplet | 48.48 | 6.63E-05 | 4 |
| Microsome | 19.19 | 1.02E-03 | 4 |
| Mitochondrion | 3.38 | 2.48E-02 | 6 |
| Membrane | 1.40 | 3.43E-02 | 19 |
| **Biological Rhythms** | | | |
| **Category** | **Enrichment** | ***P* value** | **Count** |
| Nucleus | 3.24 | 3.52E-10 | 23 |
| Cytoplasm | 2.45 | 4.28E-05 | 18 |
| **Apoptosis** | | | |
| **Category** | **Enrichment** | ***P* value** | **Count** |
| Cytoplasm | 2.50 | 6.29E-11 | 41 |
| Nucleus | 1.90 | 1.30E-04 | 30 |
| Mitochondrion | 2.53 | 1.44E-02 | 10 |
| Cytoplasmic vesicle | 3.18 | 2.05E-02 | 7 |
| Cytoskeleton | 2.19 | 4.63E-02 | 9 |
| Mitochondrion outer membrane | 6.50 | 7.57E-02 | 3 |
| **Sterol metabolism** | | | |
| **Category** | **Enrichment** | ***P* value** | **Count** |
| Microsome | 53.45 | 1.63E-12 | 9 |
| Endoplasmic reticulum | 9.77 | 3.24E-11 | 14 |
| Membrane | 1.56 | 9.62E-03 | 17 |
| Mitochondrion | 3.49 | 4.35E-02 | 5 |
| **Amino-acid biosynthesis** | | | |
| **Category** | **Enrichment** | ***P* value** | **Count** |
| Cytoplasm | 2.83 | 7.13E-02 | 4 |
| **Symport** | | | |
| **Category** | **Enrichment** | ***P* value** | **Count** |
| Cell membrane | 4.03 | 1.64E-07 | 15 |
| Membrane | 1.92 | 7.72E-06 | 19 |

**Table S10** Clinical characteristics of HCC patients.

| **Patients** | **Age** | **Gender** | **Edmonson grade** | **Tumor**  **number** | **Tumor**  **Size (cm)** |
| --- | --- | --- | --- | --- | --- |
| P1 | 36 | Male | III | 1 | 3×3×2.8 |
| P2 | 66 | Male | III | 1 | 2.5×1.5×1.3 |
| P3 | 67 | Female | III | 1 | 2.7×2.5×2.5 |
| P4 | 55 | Female | III | 1 | 6×5×4 |
| P5 | 73 | Male | II-III | 1 | 5×3.5×3 |
| P6 | 60 | Male | II-III | 1 | 6.5×5.5×3.5 |
| P7 | 49 | Male | III | 1 | 2×1.5×1.5 |
| P8 | 50 | Male | I-II | 1 | 5.5×5×4.5 |
| P9 | 61 | Male | II-III | 1 | 6.2×5×4.5 |
| P10 | 57 | Female | II-III | 1 | 7×6.5×5 |
| P11 | 71 | Male | II | 1 | 4.5×4.2×3.5 |
| P12 | 63 | Female | II-III | 1 | 8×7×7 |
| P13 | 58 | Female | II | 1 | 5×4×4 |
| P14 | 54 | Male | II | 1 | 5.5×5×3.5 |
| P15 | 57 | Male | III | 1 | 17.5×15×7.5 |
| P16 | 50 | Female | II-III | 1 | 2.3×1.8×1.7 |
| P17 | 67 | Male | III | 1 | 6.5×6×4 |
| P18 | 53 | Male | II-III | 1 | 6×4.5×4 |
| P19 | 41 | Male | I-II | 1 | 4×3.5×3.3 |
| P20 | 50 | Female | II | 1 | 1.1×0.9×0.8 |
| P21 | 51 | Male | III | 1 | 5×4×3 |

**Table S11** The metacycle analysis of POLB-interacting genes from Figure 6A.

| **CycID** | **meta2d_pvalue** | **meta2d_BH.Q** | **meta2d_period** | **meta2d_phase** | **meta2d_Base** | **meta2d_AMP** | **meta2d_rAMP** |
| --- | --- | --- | --- | --- | --- | --- | --- |
| **GOT1** | 0.0069 | 0.1789 | 24.2092 | 11.1334 | 1.6051 | 0.6532 | 0.4069 |
| **RPL23A** | 0.0083 | 0.1789 | 24.3333 | 12.2298 | 1.1519 | 0.0969 | 0.0841 |
| **RACK1** | 0.0107 | 0.1789 | 24.0887 | 22.2734 | 0.9626 | 0.1614 | 0.1614 |
| **CALR** | 0.0288 | 0.3599 | 24.3333 | 22.7763 | 0.5957 | 0.2873 | 0.2873 |
| TRIM21 | 0.0712 | 0.7116 | 23.6667 | 21.4185 | 0.8408 | 0.2197 | 0.2197 |
| UPP2 | 0.1076 | 0.8963 | 23.6667 | 9.7663 | 5.4193 | 5.1742 | 0.9548 |
| CES1D | 0.1597 | 0.9931 | 24.3333 | 11.9334 | 5.4859 | 2.7360 | 0.4987 |
| BLMH | 0.1706 | 0.9931 | 23.6667 | 1.5457 | 0.9247 | 0.1569 | 0.1569 |
| BHMT | 0.2255 | 0.9931 | 24.3333 | 22.5904 | 0.9774 | 0.2587 | 0.2587 |
| SULT1A1 | 0.3355 | 0.9931 | 23.6667 | 15.3633 | 1.1345 | 0.6925 | 0.6104 |
| PIPOX | 0.3511 | 0.9931 | 23.6667 | 9.4102 | 1.4743 | 0.3184 | 0.2160 |
| HSPE1 | 0.3789 | 0.9931 | 24.3333 | 14.8816 | 1.3205 | 0.1210 | 0.0916 |
| ATP5J | 0.3985 | 0.9931 | 23.6667 | 11.9381 | 1.2679 | 0.2652 | 0.2092 |
| FAH | 0.4286 | 0.9931 | 23.6667 | 8.7838 | 1.2065 | 0.1675 | 0.1388 |
| SAFB2 | 0.4334 | 0.9931 | 23.6667 | 10.2110 | 1.4924 | 0.4663 | 0.3125 |
| RTCB | 0.5312 | 0.9931 | 23.6667 | 2.2709 | 1.0154 | 0.1334 | 0.1314 |
| SPCS2 | 0.5313 | 0.9931 | 23.6667 | 18.7689 | 0.7862 | 0.2933 | 0.2933 |
| RPSA | 0.5551 | 0.9931 | 23.6667 | 17.0970 | 1.0378 | 0.1030 | 0.0992 |
| CYP3A11 | 0.6020 | 0.9931 | 23.6667 | 11.9418 | 1.8161 | 0.5258 | 0.2895 |
| DYNLL2 | 0.6233 | 0.9931 | 23.6667 | 11.6027 | 1.3724 | 0.6113 | 0.4454 |
| YWHAG | 0.6476 | 0.9931 | 23.6667 | 11.3718 | 1.3918 | 0.4268 | 0.3067 |
| LIMA1 | 0.6486 | 0.9931 | 23.6667 | 4.2003 | 0.9980 | 0.1786 | 0.1786 |
| ACO1 | 0.6812 | 0.9931 | 24.2071 | 10.7704 | 1.4194 | 0.1687 | 0.1189 |
| C1RA | 0.6815 | 0.9931 | 23.6667 | 3.6195 | 1.0504 | 0.1759 | 0.1675 |
| SLC25A20 | 0.7907 | 0.9931 | 23.6667 | 10.7031 | 1.3674 | 0.1455 | 0.1064 |
| NPRL2 | 0.7974 | 0.9931 | 24.3333 | 1.3743 | 1.0870 | 0.0523 | 0.0481 |
| MYL9 | 0.8056 | 0.9931 | 23.6667 | 0.7331 | 1.1646 | 0.2534 | 0.2176 |
| RPL3 | 0.8095 | 0.9931 | 23.6667 | 22.7870 | 0.9372 | 0.0409 | 0.0409 |
| MYO6 | 0.8183 | 0.9931 | 23.6667 | 10.3706 | 1.3000 | 0.1497 | 0.1151 |
| AIF1 | 0.8279 | 0.9931 | 23.6667 | 20.2358 | 0.8690 | 0.2512 | 0.2512 |
| HNRNPA2B1 | 0.8305 | 0.9931 | 23.6667 | 11.5559 | 0.9641 | 0.1379 | 0.1379 |
| C1QB | 0.8342 | 0.9931 | 23.6667 | 22.6603 | 0.7228 | 0.1613 | 0.1613 |
| SELENBP2 | 0.8469 | 0.9931 | 23.6667 | 2.5935 | 1.1654 | 0.4489 | 0.3852 |
| C1SA | 0.8877 | 0.9931 | 24.3333 | 6.4397 | 1.1403 | 0.0769 | 0.0675 |
| HAGH | 0.8916 | 0.9931 | 23.6667 | 7.2863 | 1.1781 | 0.1954 | 0.1659 |
| CMBL | 0.9171 | 0.9931 | 24.3014 | 10.7153 | 1.5438 | 0.1911 | 0.1238 |
| ARG1 | 0.9382 | 0.9931 | 24.1485 | 11.0474 | 1.0561 | 0.0767 | 0.0727 |
| SEPHS2 | 0.9424 | 0.9931 | 23.6667 | 10.2038 | 1.3790 | 0.1175 | 0.0852 |
| RPS12 | 0.9515 | 0.9931 | 23.6667 | 19.9326 | 0.9703 | 0.0555 | 0.0555 |
| SRSF9 | 0.9606 | 0.9931 | 23.6667 | 5.8141 | 1.1044 | 0.2488 | 0.2252 |
| NDUFA7 | 0.9727 | 0.9931 | 23.6667 | 3.0038 | 1.0610 | 0.0783 | 0.0738 |
| RIDA | 0.9735 | 0.9931 | 23.1047 | 9.5648 | 1.3590 | 0.1314 | 0.0967 |
| RBMXL1 | 0.9740 | 0.9931 | 23.5000 | 12.2230 | 1.0736 | 0.0523 | 0.0487 |
| MUP1 | 0.9751 | 0.9931 | 24.3333 | 2.1663 | 1.1126 | 0.2613 | 0.2349 |
| TF | 0.9774 | 0.9931 | 24.3333 | 12.6571 | 1.5482 | 0.1006 | 0.0650 |
| PCBD1 | 0.9833 | 0.9931 | 24.5000 | 7.9334 | 1.1349 | 0.0416 | 0.0366 |
| DDT | 0.9894 | 0.9931 | 24.3333 | 12.5181 | 1.2603 | 0.0017 | 0.0013 |
| PSME1 | 0.9901 | 0.9931 | 23.6667 | 5.5915 | 1.0774 | 0.1156 | 0.1073 |
| MRPS2 | 0.9922 | 0.9931 | 23.6667 | 10.5536 | 1.1209 | 0.0910 | 0.0812 |
| PRDX2 | 0.9931 | 0.9931 | 24.3333 | 0.8169 | 1.0593 | 0.0121 | 0.0114 |

**Table S12** mRNA expression levels of indicated genes annotated to the GSE133342 database.

| **Tracking_ID** | **Gene name** | **FPKM value** | | |
| --- | --- | --- | --- | --- |
|  |  | **CTL** | **Fasted 16h** | **Refed** |
| NM_010324 | *Got1* | 2636 | 16577 | 7149 |
| NM_207523 | *Rpl23a* | 6180 | 6277 | 8751 |
| NM_008143 | *Rack1* | 14881 | 12787 | 16216 |
| NM_007591 | *Calr* | 109169 | 25443 | 67704 |

**Table S13** The metacycle analysis for the circadian expression rhythmicity of CALR.

| **CycID** | **meta2d_**  **pvalue** | **meta2d_**  **BH.Q** | **meta2d_period** | **meta2d_**  **phase** | **meta2d_Base** | **meta2d_AMP** | **meta2d_rAMP** |
| --- | --- | --- | --- | --- | --- | --- | --- |
| **mRNA** |  |  |  |  |  |  |  |
| *Calr* | 6.5998e-10 | 3.2999e-09 | 24 | 2.0000 | 0.5677 | 0.2716 | 0.2716 |
| **Protein** |  |  |  |  |  |  |  |
| CALR | 0.0016 | 0.0026 | 24 | 3.6840e-05 | 0.7874 | 0.2974 | 0.2974 |

**Table S14** Sequences of primers used for RT-qPCR and BSP analysis.

| **Gene** | **Forward primer** | **Reverse primer** |
| --- | --- | --- |
| **Mouse** |  |  |
| *36b4* | 5’-GGACAGTCGATGGAACCAG-3’ | 5’-CCCTCCCACAACAAAACAA-3’ |
| *Ape1* | 5’-GCTCCGTCAGACAAAGAAGG-3’ | 5’-GCATTGGGAACATAGGCTGT-3’ |
| *Fen1* | 5’-GAACCAGGAGCAGTTTGTGG-3’ | 5’-CTCCACTTCAGCTCCACAGA-3’ |
| *Xrcc1* | 5’-ACAGTTGGAGAAGGAGGAGC-3' | 5’-AACACGGTTGGGATTTGAGC-3’ |
| *Calr* | 5’-TGGGATGAACGAGCCAAGAT-3' | 5’-TCCACTCGCCCTTGTATTCA-3’ |
| *Bmal1* | 5’-TGGAGGGACTCCAGACAT-3’ | 5’-TGGGACTTGATCCTTGG-3’ |
| *Cry1* | 5’-TACAGCAGCCACAAACAACC-3’ | 5’-GAAGCTGAGTCATGATGGCG-3’ |
| *Per1* | 5’-AACGGGATGTGTTTCGGGGTGC-3’ | 5’-AGGACCTCCTCTGATTCGGCAG-3’ |
| *Nr1d1* | 5’-ATCGTTCGCATCAATCGCAA-3’ | 5’-GTGAGGTCTCTAGAGGGCAC-3’ |
| *Dbp* | 5’-CTTTGAACCTGATCCCGCTG-3’ | 5’-TTGTTCTTGTACCTCCGGCT-3’ |
| **Human** |  |  |
| *β-ACTIN* | 5’-CACCCACACTGTGCCCATCTACGA-3’ | 5’-CAGCGGAACCGCTCATTGCCAATGG-3’ |
| *BMAL1* | 5’-AGAGGTGCCACCAATCCATAC-3’ | 5’-CCTCGGTCACATCCTACGACA-3’ |
| *CRY1* | 5’-TTGCTTGATGCAGATTGGAG-3’ | 5’-TTTTGCAGGGAAGCCTCTTA-3’ |
| *PER1* | 5’-CTGAGGAGGCCGAGAGGAAAGAA-3’ | 5’-AGGAGGAGGAGGCACATTTACGC-3’ |
| *POLB* | 5’-GAGAAGAACGTGAGCCAAGC-3’ | 5’-CGTATCATCCTGCCGAATCT-3’ |
| **MedIP primers for CpG islands of mouse *Per1* promoter and 5**'**UTR** | | |
| CpG-1 | 5’-CAAAGTATGCCCACTACGCC-3’ | 5’-TCCTTGACGACACTTACCCA-3’ |
| CpG-2 | 5’-GCGTCTCTGAGCCAATAAGC-3’ | 5’-GTTCGACGGCTCCAGAGTAA-3’ |
| CpG-3 | 5’-CCCTGAGAGCTGGTTATGGT-3’ | 5’-GAGGCCACGATACCACCTAT-3’ |
| CpG-4 | 5’-AGGAGAGGGAGGAAGTGAGA-3’ | 5’-CTCCGTGCGCACCATCAT-3’ |
| CpG-5 | 5’-CACGATGTTCCCTAGTGCG-3’ | 5’-ACAGTAACCAGGCTCTGCTG-3’ |
| CpG-6 | 5’-AGAGATCCTTAGCCAACCGG-3’ | 5’-AGCTCCGGGACAAAGACTAA-3’ |
| CpG-7 | 5’-ACAATAATCTGCCTTTCCTGTCAC-3’ | 5’-TGAAGCGGTTGACATCACGA-3’ |
| CpG-8 | 5’-GATTGGGGGAGGAGTCGTTC-3’ | 5’-ACCTGGTCTGGGACTCTGAG-3’ |
| **The BSP primer for CpG-4 island of mouse *Per1*** | | |
| CpG-4 | 5’-AGGTGGAGTTATTAAAGGATTG-3’ | 5’-CCCTATAAAATATTACCAAACCTC-3’ |
